# Methods to achieve effective web-based learning management modules: MyGJU versus Moodle

**DOI:** 10.7717/peerj-cs.498

**Published:** 2021-04-16

**Authors:** Feras Al-Hawari, Hala Barham, Omar Al-Sawaeer, Mai Alshawabkeh, Sahel Alouneh, Mohammad I. Daoud, Rami Alazrai

**Affiliations:** 1 Department of Computer Engineering, German Jordanian University, Amman, Jordan; 2 Information Systems and Technology Center, German Jordanian University, Amman, Jordan; 3 College of Engineering, Al Ain University, Abu Dhabi Campus, Abu Dhabi, UAE

**Keywords:** Software engineering, Learning managment system, E-learning, Moodle, Web application, Databases

## Abstract

Several higher education institutions have harnessed e-learning tools to empower the application of different learning models that enrich the educational process. Nevertheless, the reliance on commercial or open-source platforms, in some cases, to deliver e-learning could impact system acceptability, usability, and capability. Therefore, this study suggests design methods to develop effective learning management capabilities such as attendance, coordination, course folder, course section homepage, learning materials, syllabus, emails, and student tracking within a university portal named MyGJU. In particular, mechanisms to facilitate system setup, data integrity, information security, e-learning data reuse, version control automation, and multi-user collaboration have been applied to enable the e-learning modules in MyGJU to overcome some of the drawbacks of their counterparts in Moodle. Such system improvements are required to motivate both educators and students to engage in online learning. Besides, features comparisons between MyGJU with Moodle and in-house systems have been conducted for reference. Also, the system deployment outcomes and user survey results confirm the wide acceptance among instructors and students to use MyGJU as a first point of contact, as opposed to Moodle, for basic e-learning tasks. Further, the results illustrate that the in-house e-learning modules in MyGJU are engaging, easy to use, useful, and interactive.

## Introduction

The rapid evolution of information and communication technology significantly contributed to the adoption of web-based Learning Management Systems (LMSs) (Cavus, 2015) in higher education institutions worldwide. Such systems provide a set of powerful learning tools and are accessible anytime, anywhere. Therefore, they can empower teaching and learning by facilitating the implementation of several e-learning models (Moore, Dickson-Deane & Galyen, 2011) that satisfy the different requirements of educators and learners. Some of the e-learning models that a LMS supports are online learning (Moore, Dickson-Deane & Galyen, 2011; Sener, 2010), blended learning (Singh, 2003; Yeou, 2016), and flipped classrooms (Mok, 2014; Roach, 2014; Thai, De Wever & Valcke, 2017). Based on the study in Sener (2010), a full-scale online course utilizes only online technologies for teaching and learning. As stated in the study in Singh (2003), a blended learning experience combines offline (face-to-face classroom setting) and online (over the internet) forms of learning. According to the work in Mok (2014), in a flipped classroom the teacher records and posts lectures before class to spend the class time engaging students in interactive learning activities.

The LMSs can be categorized into three types: commercial like Blackboard (Blackboard, 2020) and Google Classroom (Abazi-Bexheti et al., 2018); open-source such as Moodle (Moodle, 2020d), Canvas (Canvas, 2020), and EDMODO (Egüz, 2020); as well as in-house as in the studies in Alseelawi et al. (2020), Aybay & Dag (2003), Bexheti, Shehu & Besimi (2009) and Jung & Huh (2019). Based on the work in Karadimas (2018), Kasim & Khalid (2016), Lawler (2011), Poulova, Simonova & Manenova (2015) and Ternauciuc & Vasiu (2015), Blackboard is the most widely used commercial LMS, and Moodle is the most popular open-source platform. Blackboard is easy to use, but it is high on pricing, does not support external plugins, and does not integrate with other platforms (Gotarkar, 2017; Poulova, Simonova & Manenova (2015)). On the other hand, Moodle is free of charge, extendable, and scalable (Gotarkar, 2017; Poulova, Simonova & Manenova (2015)). The basic LMS features were discussed in the studies in Cavus (2015), Kasim & Khalid (2016), Poulova, Simonova & Manenova (2015) and Yu, Sun & Chang (2010) and they can be grouped into three categories: resources tools such as slides, syllabus, videos, folders, and URLs; activity tools like lessons, assignments, quizzes, emails, forums, chats, and surveys; as well as administration tools including enrolments, attendance, grades, and calendar. According to the work in Borboa et al. (2017), El-Bahsh & Daoud (2016), Kc (2017), Oproiu (2015) and Yeou (2016), the resources tools are the most utilized, as opposed to the other tools, by LMS users.

In particular, the Moodle LMS has been used at the German Jordanian University (GJU) as an e-learning platform since 2010. In 2015, the ineffective commercial Student Information System (SIS) was replaced with an in-house SIS named MyGJU Admin (Al-Hawari et al., 2017), a Human Resources (HR) system, and an Accounting Information System (AIS) (Al-Hawari, 2017a; Al-Hawari & Habahbeh, 2020). Moreover, the in-house university portal named MyGJU (Al-Hawari, 2017b; Al-Hawari et al., 2017) has been released to permit instructors to access needed information such as schedules, publications, commissions, and salaries. MyGJU also allows students to view academic information like majors, study plans, course sections, grades, and advisors. It also lets students view financial data such as scholarships, tuition fees, and registration invoices.

Accordingly, the SIS database became available to the development team and thus allowed uploading course sections and student enrolments to Moodle via the external database enrolment plugin (Moodle, 2020b). Despite the attained ability to easily synchronize some of the SIS data with Moodle, the fact that users still had to use two partially-integrated platforms to perform daily academic tasks remained ineffective for the following reasons:

- Depending on two e-learning platforms sometimes forces users to repeat the work done in one platform in its counterpart in order to accomplish basic tasks. For example, an instructor needs to redefine the already defined course days in MyGJU to setup the attendance module in Moodle. Besides, an instructor needs to enter the student grades in a certain quiz in Moodle and then in MyGJU to account for them in the course official mark.
- Second party LMSs may possess serious structural problems. For example, Moodle adopts a flat (non-hierarchical) course-based structure that does not allow organizing courses according to faculties, degrees, majors, or semesters. Accordingly, a naming scheme for courses must be enforced to allow finding courses that are repeatedly offered every semester. Furthermore, deans cannot easily manage the resources of their schools for either evaluation, approval, analysis, or reporting.
- Second party LMSs may have annoying usability issues. For example, Moodle has no single user-friendly solution for sharing resources between courses. The choice to make the resources available to any course by placing them in the site files directory is non-secure as they can be accessed by anyone. The meta-course module is not easy to manage and only allows sharing files between courses of the same instructor. That hinders the coordination among instructors and leads to consuming more storage space because any overlapping content has to be duplicated in all the same courses that are assigned to different instructors. Other solutions require having admin rights, which is not the case of instructors.
- Customizing an open-source LMS to meet GJU requirements and standards requires advanced programming skills and is not trivial.

In that respect, this paper discusses the basic learning management modules that have been recently offered in MyGJU to support online learning. Specifically, important capabilities such as attendance, emails, coordination, course information, course folder, syllabus, course section homepage, student tracking, and survey are released to make MyGJU, rather than Moodle, a First Point of Contact (FPOC) for educators and students to accomplish basic e-learning tasks. Nevertheless, several mechanisms have been also proposed to overcome the aforementioned challenges that were encountered when using Moodle for e-learning. Particularly, ways to facilitate system setup, information reuse, version control automation, manager collaboration, and feature customization have been suggested to make the tools efficient and engaging, hoping the result of that would motivate both educators and students to take part in online learning. Noting that other features in MyGJU such as profile, course sections, registration, schedules, study plans, transcripts, and fees were discussed in the study in Al-Hawari (2017b). Besides, the MyGJU administration related options like buildings, faculties, majors, semesters, calendars, user roles, admission, major transfers, grades processing, graduation, and reports were shown earlier in the work in Al-Hawari et al. (2017).

The rest of the paper is organized as follows. In “Literature Review”, a literature review is presented. In “Project Management and E-Learning Modules Design”, the needed project management and software design methods related to the required e-learning features in MyGJU are introduced. In “Methods to Overcome the Moodle Drawbacks”, factors such as direct database access, information reuse, data version control, and role-based user collaboration that primarily permitted MyGJU to overcome the Moodle drawbacks are emphasized. In “Discussion”, the effectiveness of the e-learning modules in MyGJU over their counterparts in Moodle, the limitations of MyGJU compared to Moodle, and a comparison between MyGJU and existing LMSs are discussed. The validation results for the new features are shown in “Validation and Results”. Finally, conclusion and future work are provided in “Conclusion and Future Work”.

## Literature review

The utilization of e-learning to improve the learning experience has attracted researchers and educators in the past years. However, according to the study in Ali, Uppal & Gulliver (2018), there are several barriers that could impact the success of e-learning implementations. The identified barriers were grouped into the following four categories: technology, individual, pedagogy, and enabling conditions. The technology barriers are related to software interface design, hardware infrastructure, network bandwidth, internet connectivity, quality of equipment, and technical support. Examples on the individual barriers are: attitude towards ICT, computer literacy, cost of using technology, and student readiness. Some of the pedagogy related barriers are: faculty effort, faculty training, quality of course content, engaging students online, capabilities of the LMS, and real-time feedback. The enabling conditions are tied to administrative support, setup cost, rules and regulations, language barriers, load shedding of electricity, as well as ethical barriers.

Similarly, the key issues associated with the implementation of LMSs were addressed in the study in Turnbull, Chugh & Luck (2021). Some of the identified themes were related to LMS selection and non-financial factors, as well as to learner and faculty impressions of online learning. Accordingly, decision makers often rate cost as one of the most important factors in selecting a LMS. For commercial systems, these costs could include software licensing and provider services charges. On the other hand, the costs for in-house or open-source solutions are more dependent on IT staff expenses during the projects lifespan. However, reliance on financial factors alone could preclude choices with better functionality. As far as the learner and faculty impressions of online learning, some teachers were concerned about the capacity for students to embrace self-study approaches to learning. Whereas, the faculty skills and adopted technologies were identified as important factors that may impact upon the motivation of students to engage in online learning. Likewise, the study in Frank & Barzilai (2008) concluded that technology should be used to enable active learning, provide prompt feedback, and make use of multimedia representation. In addition, it was found that instructors tend to not make use of the full technology potential when building course pages.

In that respect, several institutions have implemented their own LMS in-house to better integrate with their instructional resources or to cut costs (Yueh & Hsu, 2008). For example, the study in Alomran et al. (2020) introduces an effective e-learning platform that offers various e-learning modules such as attendance, assignments, quizzes, surveys, grades, and messages. An n-tier system that supports sharing and revising learning materials is presented in Alseelawi et al. (2020). The study in Jung & Huh (2019) proposed a LMS that enables learners to apply for, and to take, online courses. It also suggested hosting the LMS in a cloud-based test bed that is efficient, reliable, and secure. The design and implementation of a mobile learning system named Easy-Edu are shown in the study in Elkhateeb, Shehab & El-Bakry (2019). The system includes areas for discussion, learning materials, questions, feedback, and announcements. The LMS introduced in Abdulazeez & Zeebaree (2018) consists of nine user views that provide services to students, lecturers, departments, and university. It supports advanced user roles such as head of department, QA user, exam committee user, and curriculum development admin. It also offers basic e-learning services such as lectures, assignments, grades, and forum. In the study in Wu et al. (2018), the WeChat API is utilized to develop a college online learning system that consists of a learning module, test module, and information management module. A system named CEIBA is discussed in the study in Yueh & Hsu (2008). It is implemented in-house to focus on the teaching aspects of using an LMS. It also supports features such as syllabus, weekly learning materials homepage, files, merging courses, importing course content, assignments, and interactive activities. The study in Aybay & Dag (2003) introduces a LMS that allows tracking student activities as well as managing courses, lectures, assignments, quizzes, and announcements.

Recently, the concept of gamification has been investigated in the context of education and training as it offers several advantages associated with learning outcomes (Osatuyi, Osatuyi & De La Rosa, 2018). Gamification has been defined as *the use of game design elements in non-game contexts* according to the research in Deterding et al. (2011). In particular, an online learning management with gamification is introduced in the study in Liu (2020) with the hope of making the learning experience more engaging and enjoyable. The LMS incorporated gamification elements such as game map, virtual currency, items, narrative, story line, virtual store, user inventories, and tasks. It laid out lessons and quizzes on a game map to allow learners (players) to defeat (complete) enemies (quizzes) in a game-like manner. The quizzes include randomized narratives to make the learner feel that an enemy is defeated or a contract is completed. The learner (player) is randomly rewarded virtual currencies when quizzes (enemies) are completed (defeated). The player may use the virtual currency to buy items from the virtual store, then the purchased items will be placed in the player’s inventory.

Further, based on the study in Mershad et al. (2019), the Internet of Things (IoT) is expected to be the most important technology to affect the structure, operations, and implementation of LMSs. Accordingly, an IoT-enhanced LMS may improve the learning process in several aspects such as experimentation, virtual reality, remote lectures, data sharing, student assessment, security, classroom applications, and classroom monitoring. Besides, the use of artificial intelligence (AI) in LMSs has been investigated in the work in Aldahwan & Alsaeed (2020). In that aspect, AI approaches can be useful to provide features such as chatbots based on natural language processing (Murad et al., 2019), recommender systems (Marante et al., 2020), and learning analytics (Viberg et al., 2018).

Therfore, this work focused on identifying the basic software modules to support e-learning in a university portal, with specific emphasis on techniques to eliminate any barriers that could impact the successful deployment of such features. In particular, it proposed mechanisms to simplify system setup, enable information reuse, automate version control, and support advanced user roles. Based on that, the hope is to achieve an easy to use and engaging system that would motivate both educators and students to take part in online learning. Correspondingly, the main advantages of the proposed e-learning modules in MyGJU as opposed to their counterparts in Moodle as well as the in-house LMSs in the studies in Abdulazeez & Zeebaree (2018), Alomran et al. (2020), Alseelawi et al. (2020), Aybay & Dag (2003), Elkhateeb, Shehab & El-Bakry (2019), Jung & Huh (2019), Wu et al. (2018) and Yueh & Hsu (2008) are emphasized in “Discussion”. Not to mention, machine learning approaches (Al-Hawari & Barham, 2019), IoT technologies (Al-Hawari, Al-Sammarraie & Al-Khaffaf, 2020), and gamification elements like badges are being investigated to be utilized in MyGJU for learning purposes.

## Project management and e-learning modules design

Various process and design related topics pertaining to the development of the e-learning features in MyGJU are covered in this section. Initially, the adopted project management process and software development cycle are discussed. Then, the e-learning features provided to the different user roles are specified. Next, the entity relationships (ER) diagrams for the database tables of the suggested e-learning features in MyGJU are presented. Finally, the different modules in the multiple layers of the MyGJU three-tier system architecture are explained.

### Project management and development processes

The MyGJU project releases are managed according to the project management steps that are suggested in the systems engineering basic profile in the ISO/IEC 29110 series (Laporte, O’Connor & Paucar, 2015). The adopted project management process encompasses the following six important steps: project plan, progress reports, review meetings, version control, risk management, and disposal plan. Furthermore, each e-learning module is considered as an increment in the utilized iterative and incremental software development process that proposes using repeated waterfall cycles (iterations) to develop each software module (increment) in the system.

### E-learning features specifications

**Figure 1:**
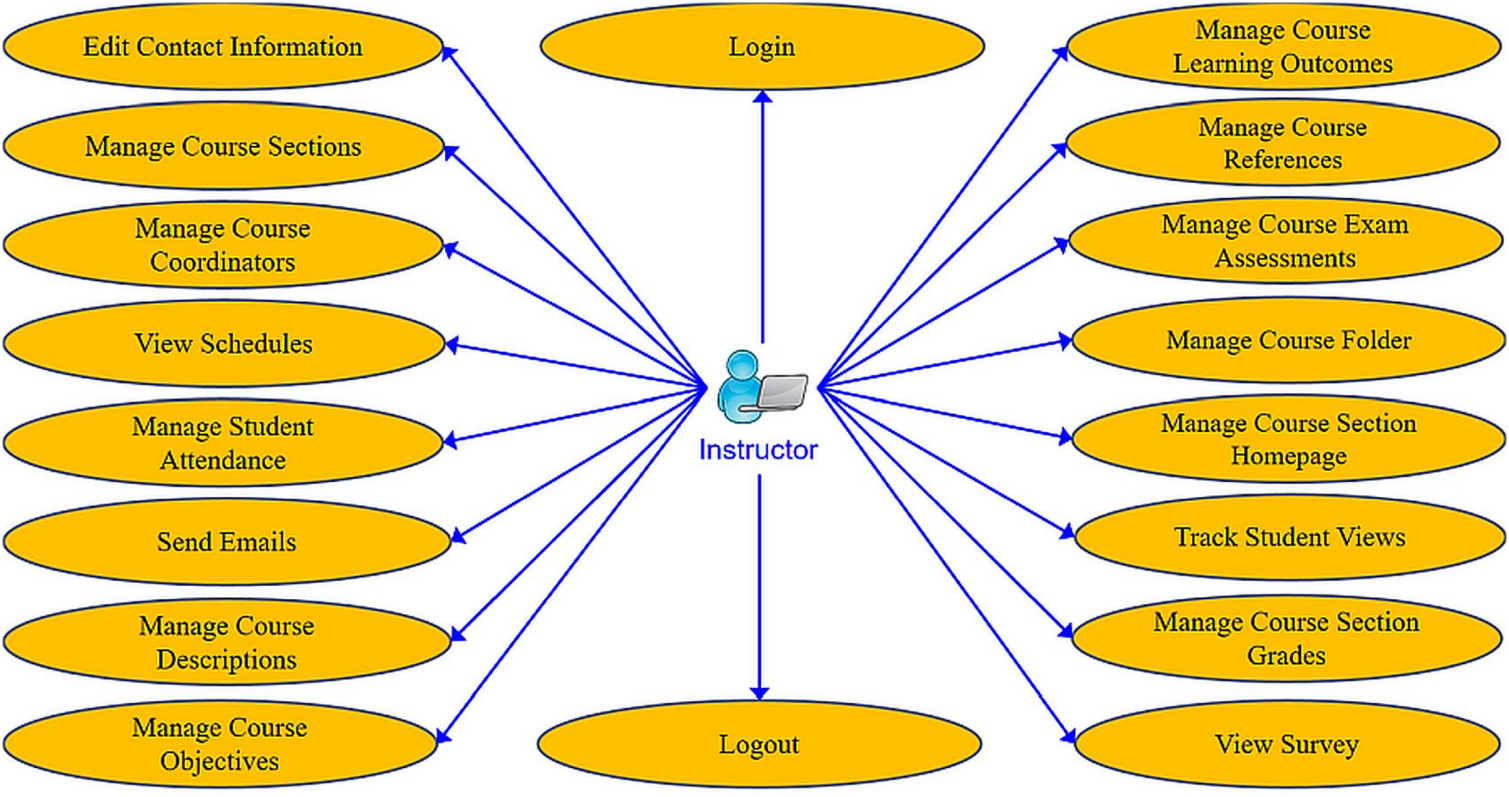
The use case diagram for the basic e-learning features in the MyGJU instructor view.

**Figure 2:**
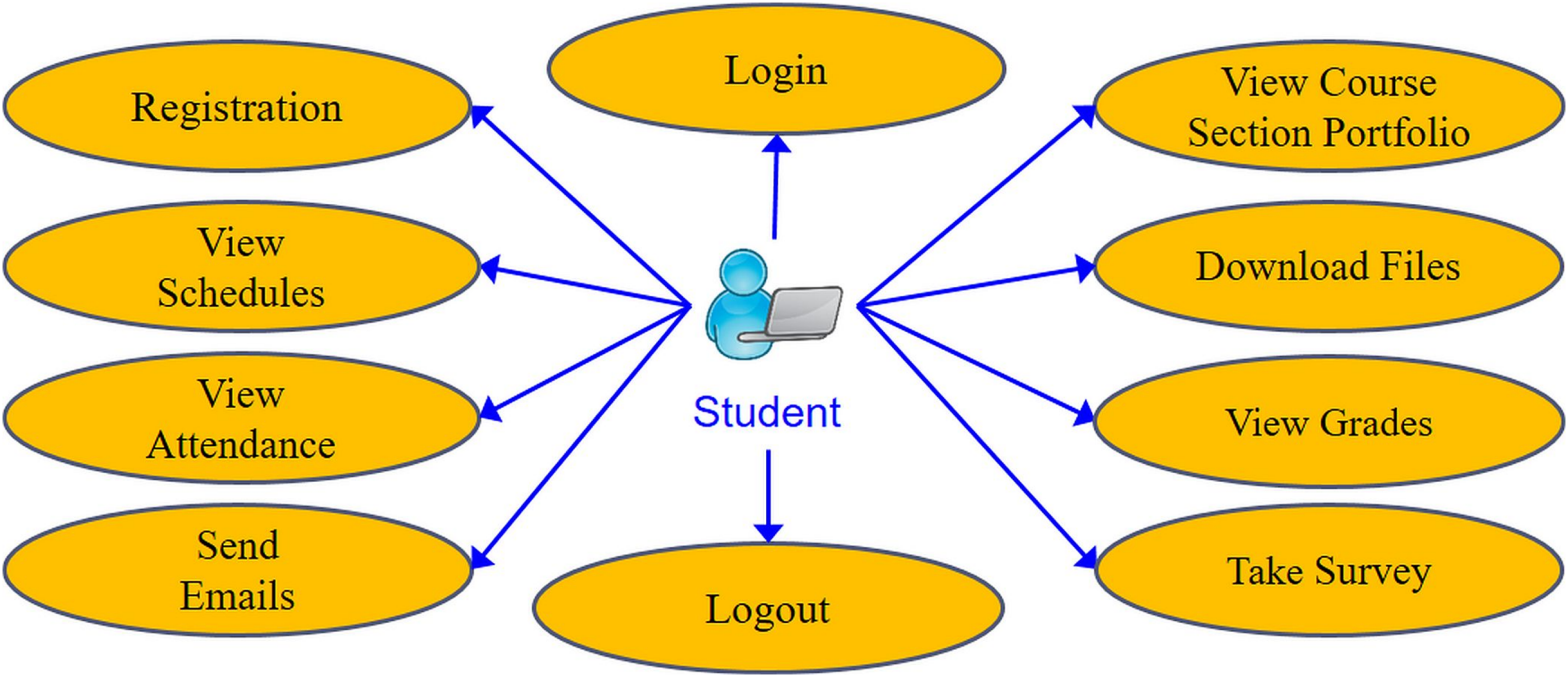
The use case diagram for the basic e-learning features in the MyGJU student view.

The e-learning features that were developed in MyGJU to replace their Moodle counterparts are specified in this section. The instructor-centered features and student-related features are summarized in Figs. 1 and 2, respectively. The e-learning features that are accessible from the instructor view in MyGJU (Al-Hawari et al., 2017) are discussed next. Noting that the features in the student view (Al-Hawari, 2017b) are not considered further as they are very similar to the features in the instructor view, but in most cases the student has view rather than management access to such capabilities.

**Figure 3:**
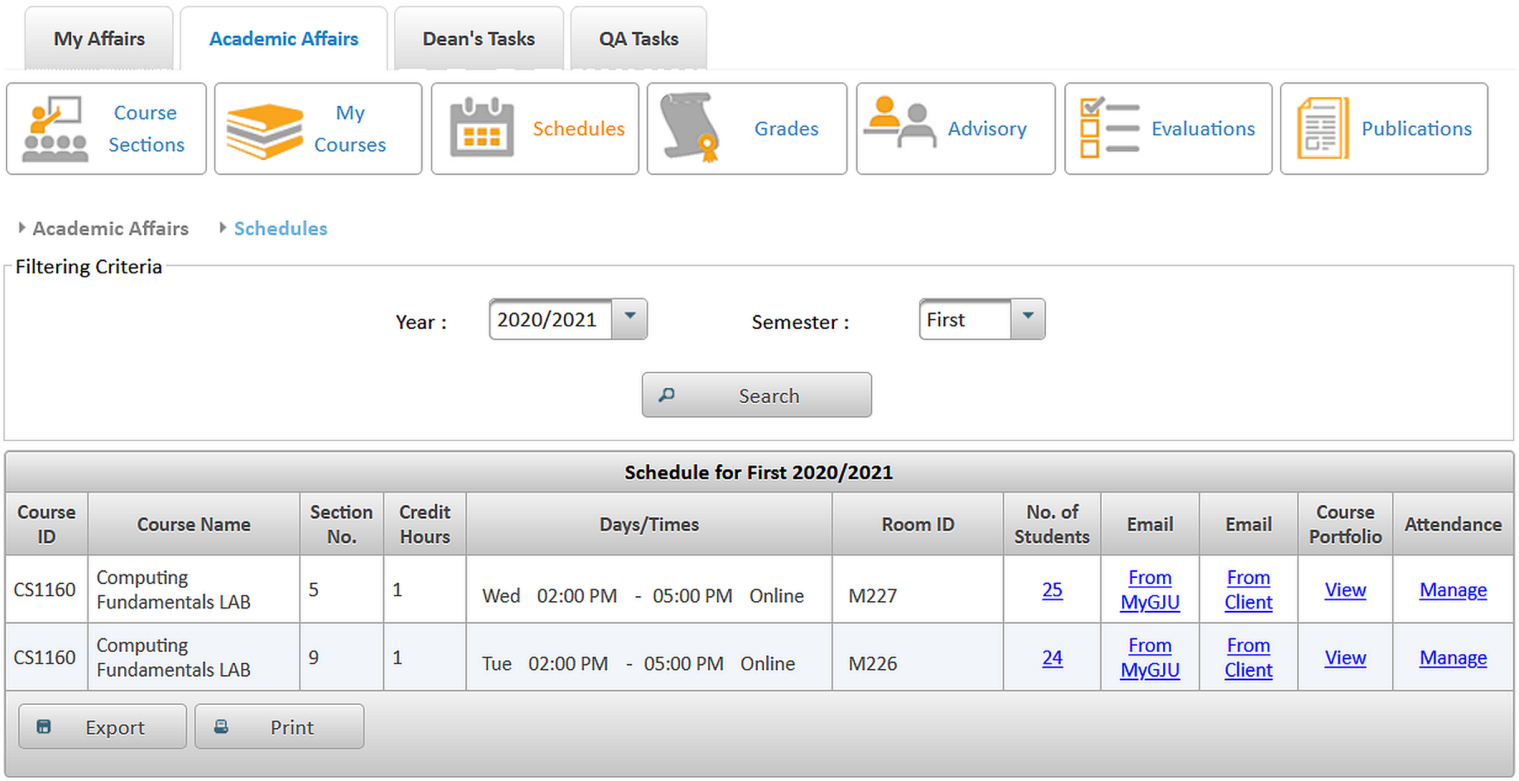
The instructor course section schedule screen.

**Figure 4:**
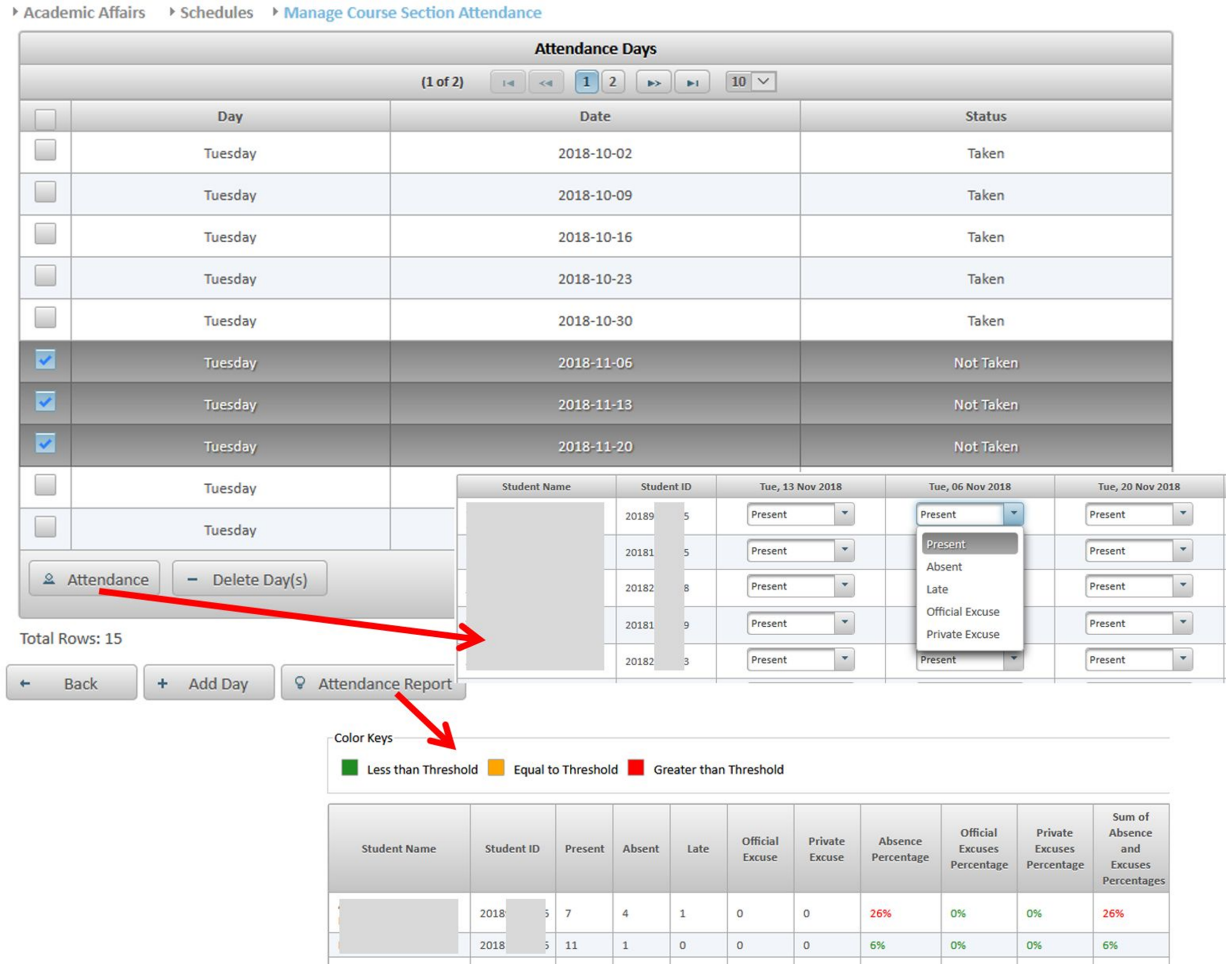
The various screens in the manage student attendance flow.

**Figure 5:**
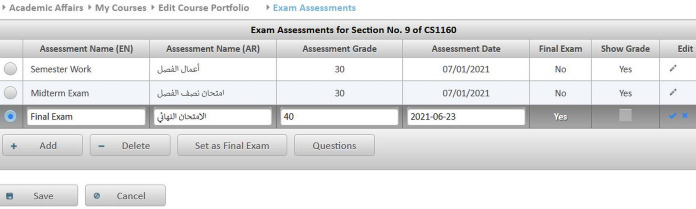
The manage exam assessments screen.

**Figure 6:**
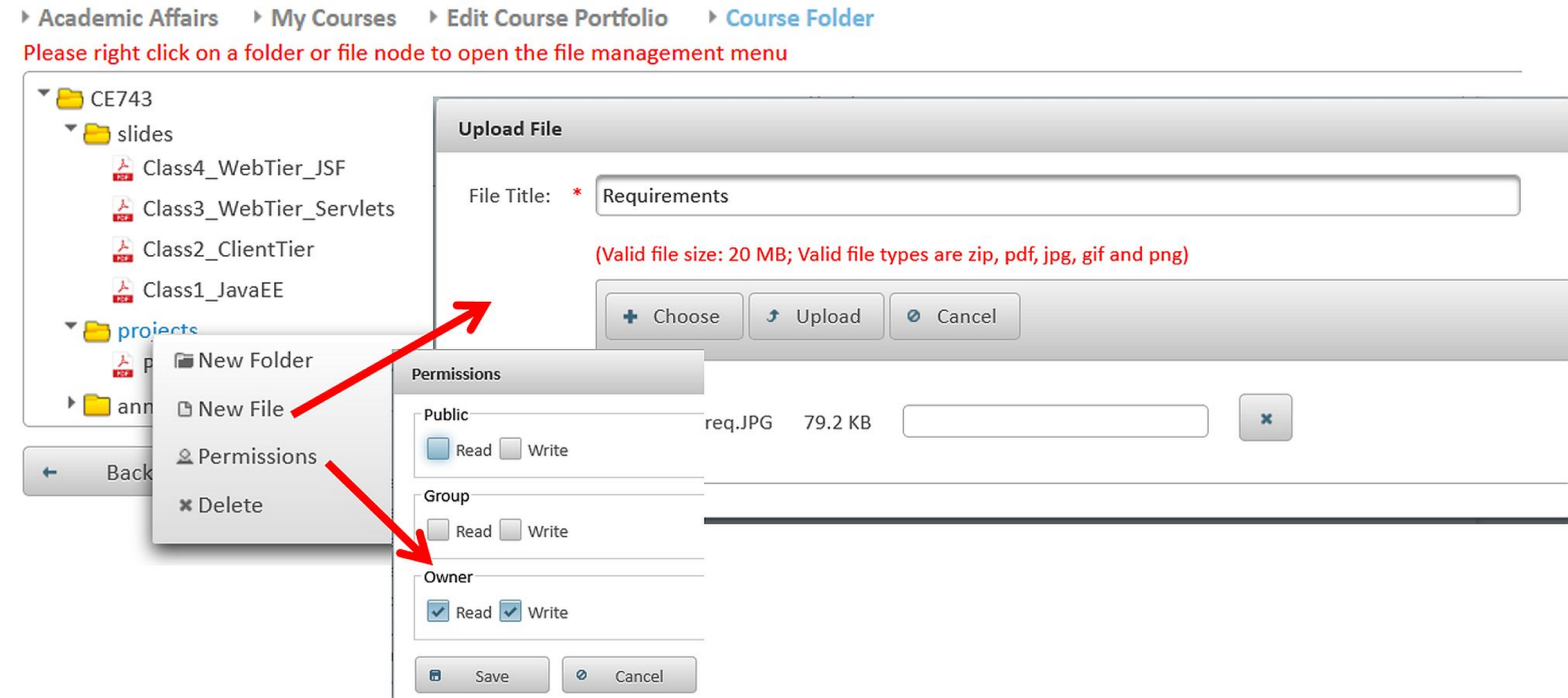
The course folder for course CE743.

**Figure 7:**
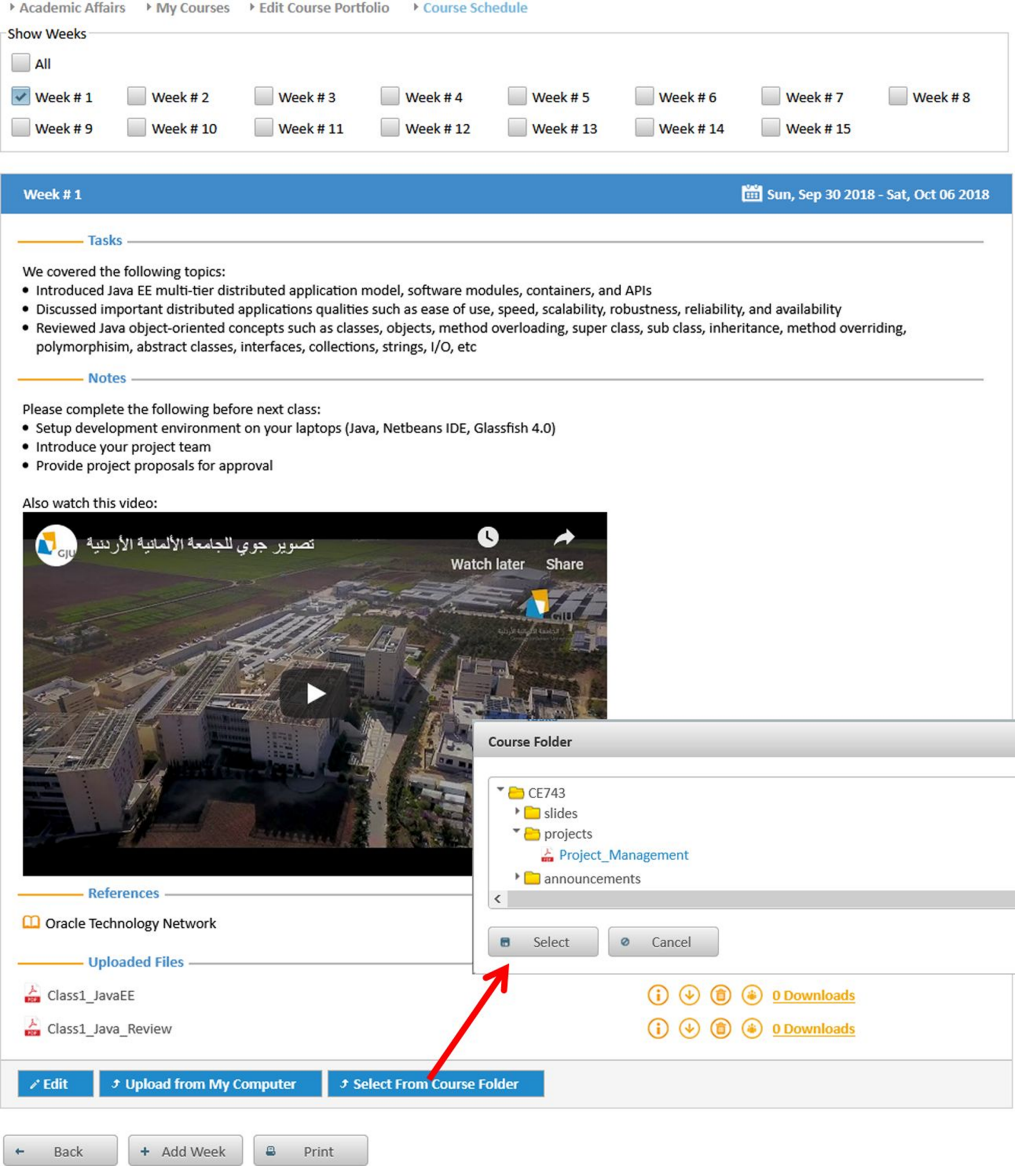
The edit course section weekly learning materials part in the course section homepage.

**Figure 8:**
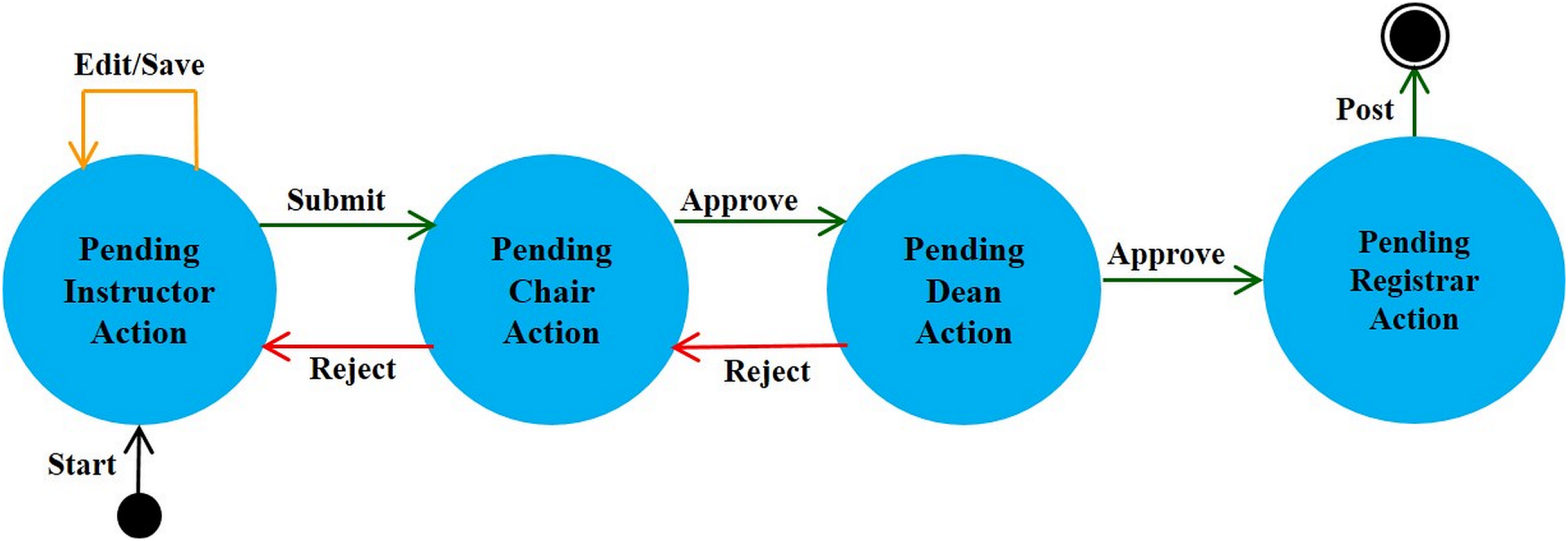
The grades submission state diagram.

**Figure 9:**
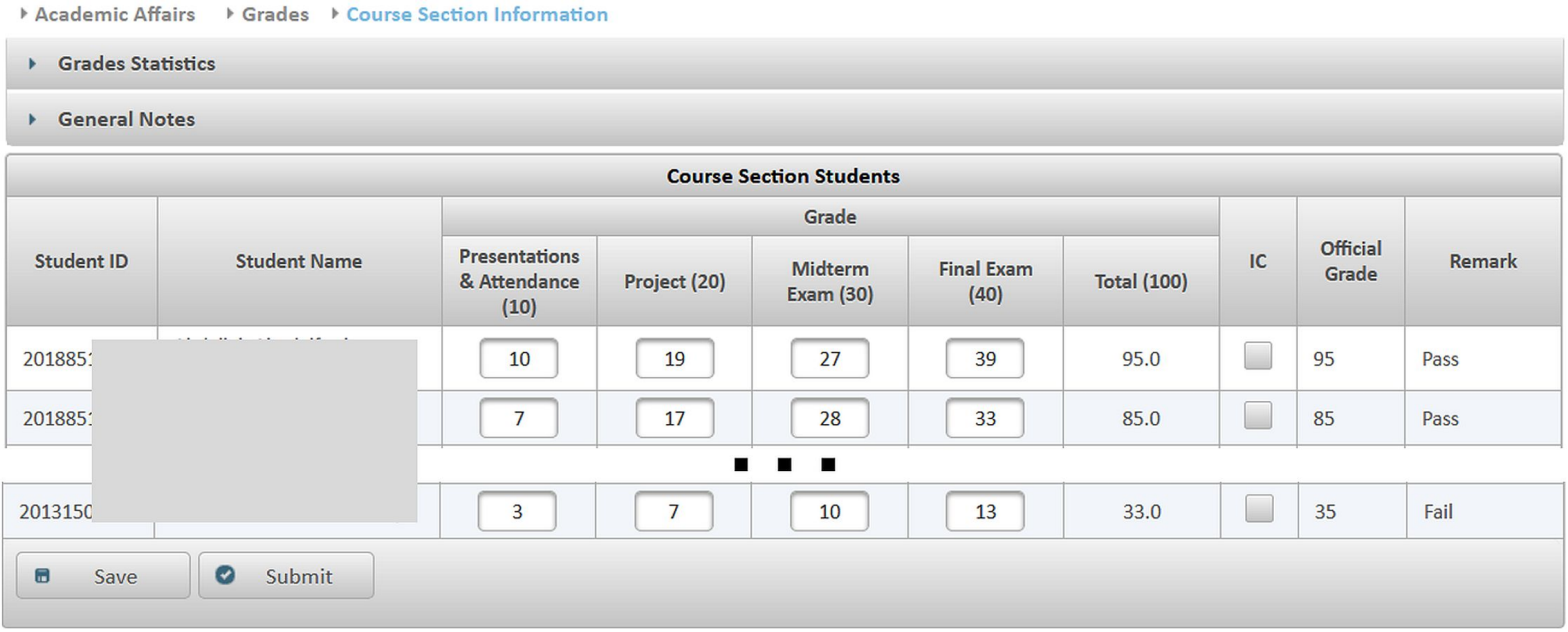
The grades submission screen.

1. **Editing contact information:** Instructors may update their contact information such as office location, phone number, office hours, and webpage to be automatically posted in relevant pages like the course syllabus and course section homepage.
2. **Managing course sections:** As discussed in Al-Hawari et al. (2017), instructors with either a dean or a chair role can use MyGJU to manage the course sections offered by their departments each academic semester. In this context, a user with data management privilege can add, edit, view, or delete the related information.
3. **Managing course coordinators:** Instructors with either a dean or chair role can assign/unassign course coordinators. A course coordinator is responsible for building weekly learning materials that will be reused in all shared course sections.
4. **Viewing instructor course schedules:** MyGJU lets instructors view their course schedule for any semester (see Fig. 3). Besides, instructors can view basic information like photos, names, and majors of students enrolled in their courses. Not to mention, the course schedules of the instructors in a school or department are accessible to the school dean or department chair, respectively, to facilitate administrative tasks such as scheduling meetings and assigning tasks.
5. **Managing student attendance:** The system offers a flexible student attendance management flow (shown in Fig. 4) that supports the following functionalities: automatic generation of attendance days based on the course section schedule and semester period; ability to take student attendance for multiple days at once; option to select from various attendance states such as present, absent, late, official excuse, and private excuse; as well as automatic determination of student attendance violations based on GJU regulations for further action by the instructor, for example dismissing a student from the course.
6. **Sending Emails:** Instructors can send emails to their students from MyGJU or the email client. For example, the system automatically builds the recipient list of the email message based on the usernames of the students enrolled in a course section. Moreover, it easily allows a dean or a chair to customize the recipient list based on different filtering criteria such as student ID, degree, major, enrollment year, and enrollment status.
7. **Updating course descriptions:** An instructor can update the description of a related course to summarize the benefits and learning experiences that the students may anticipate.
8. **Managing course objectives:** The objectives of a course can also be managed by the related instructors to make the course aims clear to the students.
9. **Managing course references:** An instructor can further manage the references list for a course. A reference in this context can be either a textbook, article, or URL. Based on that, students can use the listed references to acquire the needed materials to study for the course.
10. **Managing course learning outcomes:** Each course is associated with learning outcomes to determine what the students know, understand, and are capable of after completing the course. Accordingly, a learning outcome measures one of the following learning methods: knowledge, skills, or competencies. Consequently, each question in an exam assessment can be used to evaluate one or more course learning outcomes. Based on that, the system can automatically estimate what the students learned from a course based on their exam results.
11. **Managing course exam assessments:** The exam assessments of a course section can be managed (see Fig. 5) to serve as a grading guide that enables students to know the weighting of each assessment. Thus, an instructor can specify the name, grade, and date of each assessment. Besides, the final exam assessment needs to be identified to enable the system to publish the pre-final grades before the start of the final exams, and hence comply with the university regulations. Also, the Show Grade checkbox is used to hide/show the related assessment grades from/to the students.
12. **Managing course folder:** Each course is associated with a folder as shown in Fig. 6 to be used by instructors for uploading and organizing course materials such as slides, exercises, homework, notes, and videos. The system supports uploading files as large as 20 megabytes to a folder, and it has no limit to the number of uploaded files. It also scans each file for viruses to prevent uploading infected files to the server. Once a file is uploaded, it can be linked to the weekly learning materials homepages of several course sections to make it available to the students. Hence, uploading a file once and reusing it many times improves usability and conserves storage. Moreover, the uploaded files can be organized into subfolders with meaningful names. Nevertheless, the system also supports file and subfolder permissions (see Fig. 6) that allow instructors to control who can read or write their files and subfolders. The read access permits downloading a file and viewing the contents of a subfolder. Whereas, the write access allows deleting and renaming files and folders, besides adding files and subfolders to a folder. The allowed permission groups are owner (the instructor who added the file), group (dean, chair, and coordinator), and public (all course instructors). It is also worth noting that due to privacy and copyright concerns, only the owner of a file, or a subfolder, is authorized to change its access permissions to make it available/hidden to/from other instructors.
13. **Managing course section homepage:** A course section homepage is split into the following parts: header, instructor information, course information, learning outcomes, exam assessments, and weekly learning materials. The learning materials part is organized on a weekly basis. Accordingly, the system automatically generates one division per week in the defined teaching period for the semester. Consequently, an instructor can post relevant tasks, notes, references, and files within each week’s division (see Fig. 7) in the homepage. In that regard, the tasks and notes can be edited using a rich text editor that supports useful capabilities such as formatting style, checking the spelling, embedding videos, inserting images, adding webpage links, and defining mathematical equations using latex. Besides, the week references can be conveniently chosen from the predefined references list of the course. Also, the files can be either uploaded from the instructor’s computer or reused from the shared folder of the course.
14. **Managing course section grades:** Instructors may manage grades throughout the grade submission period (Al-Hawari et al., 2017) for a semester. In that regard and according to the grade submission state diagram shown in Fig. 8, an instructor starts entering and saving the exam assessment grades for each student in a course section (see Fig. 9). After the grades are entered, the instructor submits them for approval by the department chair and later by the school dean. It is worth noting that an instructor cannot edit the grades after submitting them unless they get rejected by the chair, or by the dean and then by the chair, for corrections. Moreover, the grades are considered official when approved by the dean and therefore the relevant registrar may then post them to the students' official transcripts (Al-Hawari et al., 2017).
15. **Viewing surveys:** Before the completion of a course, the enrolled students can take a survey to evaluate the performance of their instructors. In turn, instructors can view their related scores for reference. Besides, the scores of instructors in a faculty or department are accessible to the school dean or department chair, respectively. Based on that, the schools and departments can assess the performance of their instructors, improve the educational processes, and enhance the content of the courses.
16. **Tracking student views:** Instructors can view student tracking details for their course section homepages. Specifically, they can view information such as who accessed a file or homepage, when a file or homepage was viewed, and how many times a user accessed a file or homepage.

### Database design

**Figure 10:**
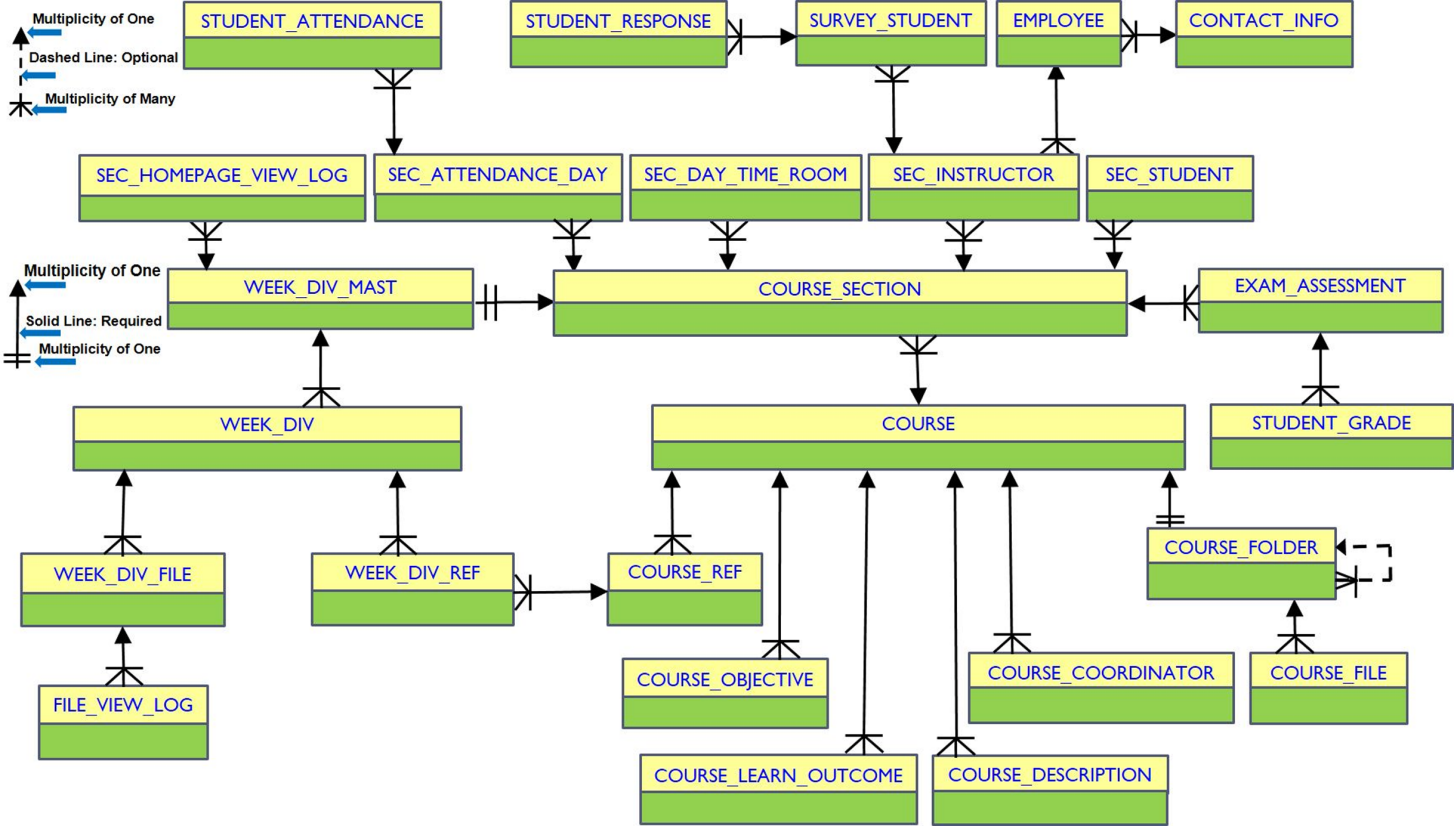
The ER diagram for the database tables of the e-learning modules.

The ER diagram for the database tables of the e-learning modules in MyGJU is shown in Fig. 10. Accordingly, a course in the COURSE table can be associated with course sections in the COURSE_SECTION table, descriptions in the COURSE_DESCRIPTION table, objectives in the COURSE_OBJECTIVE table, learning outcomes in the COURSE_LEARN_OUTCOME table, references in the COURSE_REF table, coordinators in the COURSE_COORDINATOR table, and a course folder in the COURSE_FOLDER table. A course folder can be associated with child course folders and files in the COURSE_FOLDER table and COURSE_FILE table, respectively.

The course sections days, times, and rooms are stored in the SEC_DAY_TIME_ROOM table. A course section is assigned to instructors in the SEC_INSTRUCTOR table, whereas the contact information for the instructor teaching a course section are saved in the CONTACT_INFO table. The students enrolled in a course section are recorded in the SEC_STUDENT table. Yet, each course section can be associated with weekly learning materials through the WEEK_DIV_MAST table. The weekly learning materials are associated with weeks stored in the WEEK_DIV table. A week may be associated with several files and references in the WEEK_DIV_FILE table and WEEK_DIV_REF table, respectively. Each reference in the WEEK_DIV_REF table is associated with a course reference in the COURSE_REF table. When a course section homepage or a file in a week division is viewed by a user, a related transaction is logged in the SEC_HOMEPAGE_VIEW_LOG table or FILE_VIEW_LOG table, respectively.

A course section can also have several exam assessments in the EXAM_ASSESS table. The student grades in each exam assessment are saved in the STUDENT_GRADE table. Not to mention, the attendance days associated with a course section are generated in the SEC_ATTENDANCE_DAY table, while the taken attendance operations for all students in a course section are recorded in the STUDENT_ATTENDANCE table. Besides, students who participated in the survey to evaluate a course section instructor are recorded in the SURVEY_STUDENT table, whereas the student responses to the survey questions are saved in the STUDENT_RESPONSE table.

**Figure 11:**
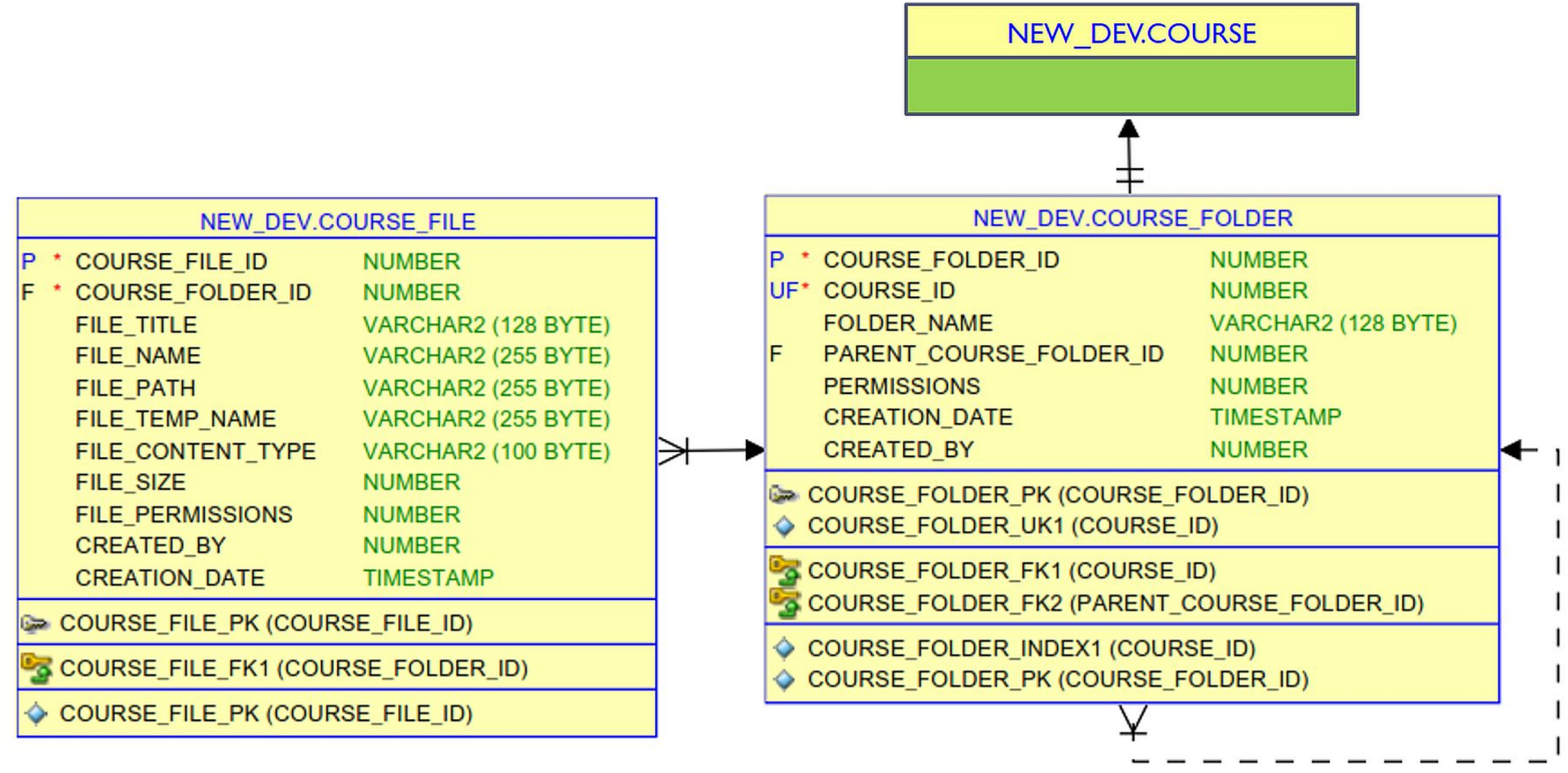
The detailed ER diagram for the course folder management tables.

Further, the detailed ER diagram for the database tables that are used by the manage course folder feature, for example, is shown in Fig. 11. Accordingly, each course in the COURSE table is associated with one course folder in the COURSE_FOLDER table. A course folder can contain files and subfolders. The COURSE_FILE table stores file meta-data such as title, name, path, temp name, content type, size, and permissions. Whereas, the COURSE_FOLDER tables contains the course ID, folder name, permissions, and the ID of the parent folder.

### Three-tier system architecture

**Figure 12:**
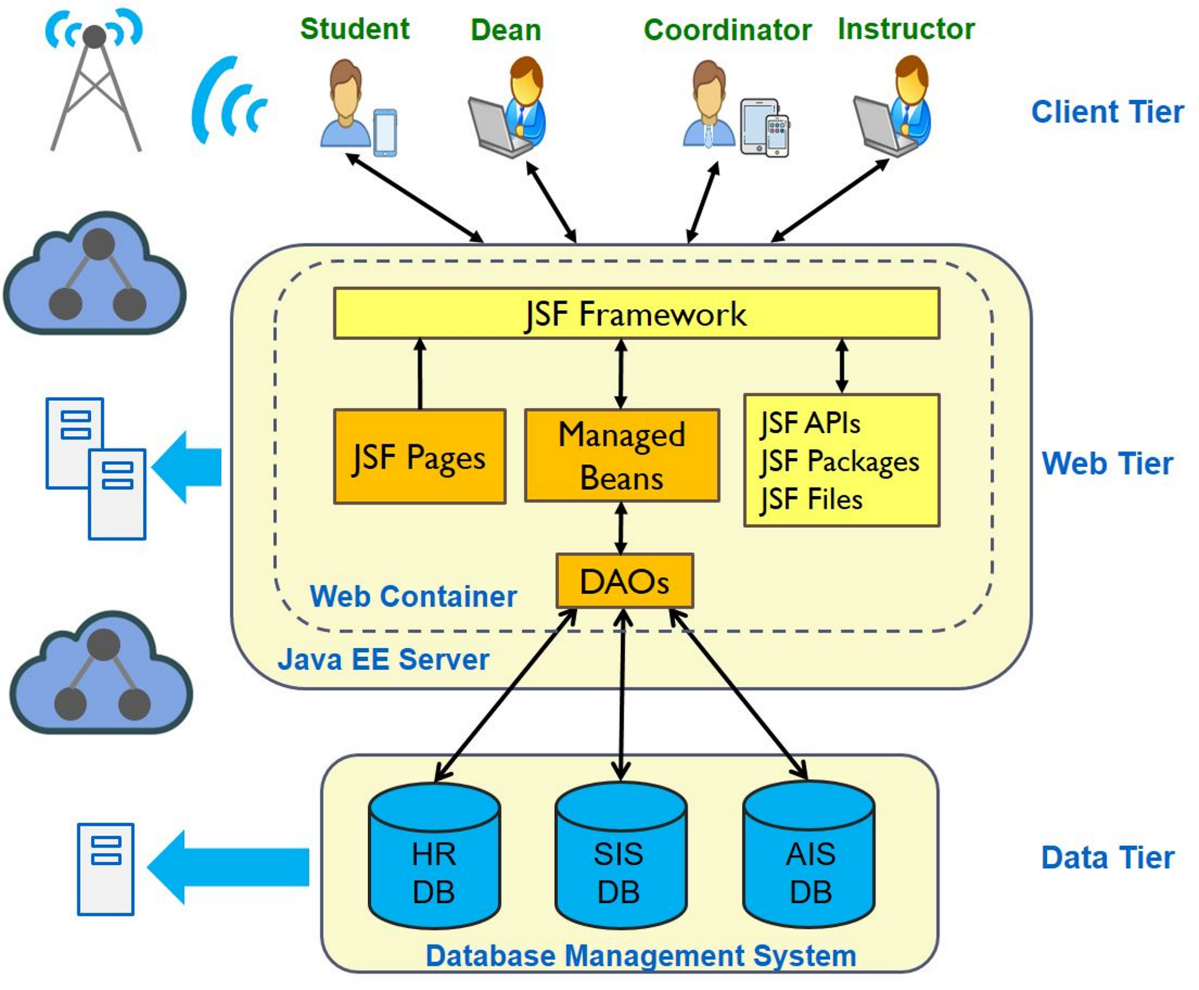
The MyGJU three-tier system architecture.

The MyGJU portal is a web-based Java Enterprise Edition (EE) application that is implemented according to the three-tier architecture shown in Fig. 12. The adopted three-tier architecture is a client-server based architecture that allows the physical separation of the user interface, application logic, and data management layers across client, web, and business tiers, respectively. Accordingly, a web browser running on the clients’ devices such as personal computers and mobile phones is required to access the e-learning modules in the client tier. Besides, the Java EE web-application server is utilized to host the different e-learning software modules in the web (application) tier. Further, the Oracle Relational Database Management System (RDBMS) is used to administer the SIS, HR, and AIS databases (shown in Fig. 12) in the business tier.

An e-learning module is comprised of JavaServer Faces (JSF) pages, Java beans, and Data Access Objects (DAOs). A JSF page contains HTML, JSF, and Primefaces elements to represent the Graphical User Interface (GUI) components in the corresponding screen. The JSF and Primefaces packages support many GUI components such as menus, text fields, checkboxes, tables, calendars, and slideshows. Not to mention, the JSF framework also provides packages for data validation, data conversion, event handling, templates, and internationalization. Whereas, a Java bean is a special Java class that is managed by the web container, in other words the web container is responsible for instantiating, executing, updating, and destroying the managed Java bean. The main responsibilities of a Java bean are to save the state of the corresponding JSF page and to handle the application logic of the page. Yet, a DAO is used by a Java bean to access/update data from/in the databases in the business tier. Specifically, it uses the Java Database Connectivity (JDBC) API to interact with the databases in the Oracle RDBMS.

## Methods to overcome the moodle drawbacks

The factors that primarily enabled e-learning modules in MyGJU to overcome the drawbacks of their counterparts in Moodle are emphasized in this section.

### Direct access to the university databases

**Figure 13:**
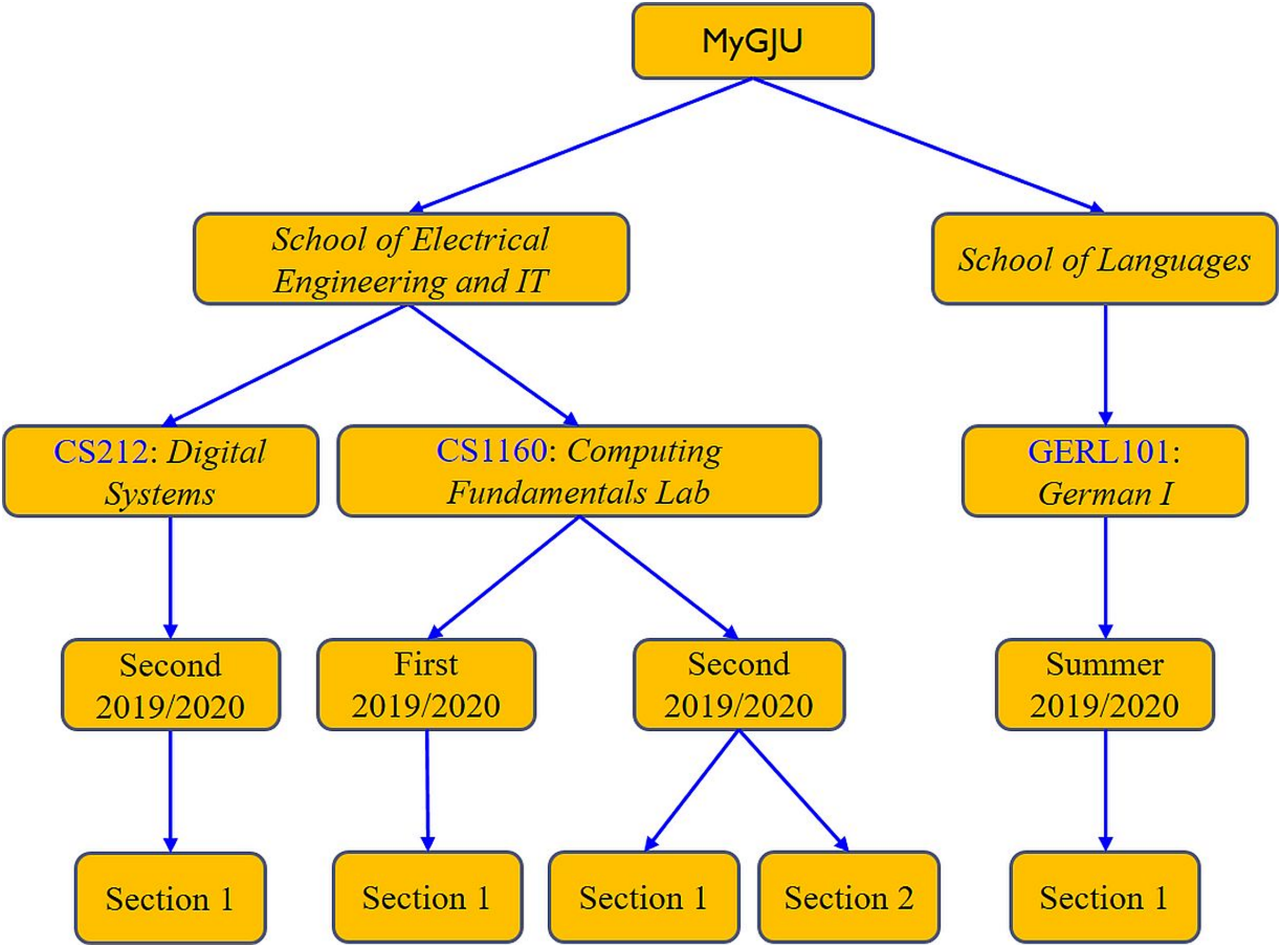
The hierarchical course structure in MyGJU.

**Figure 14:**
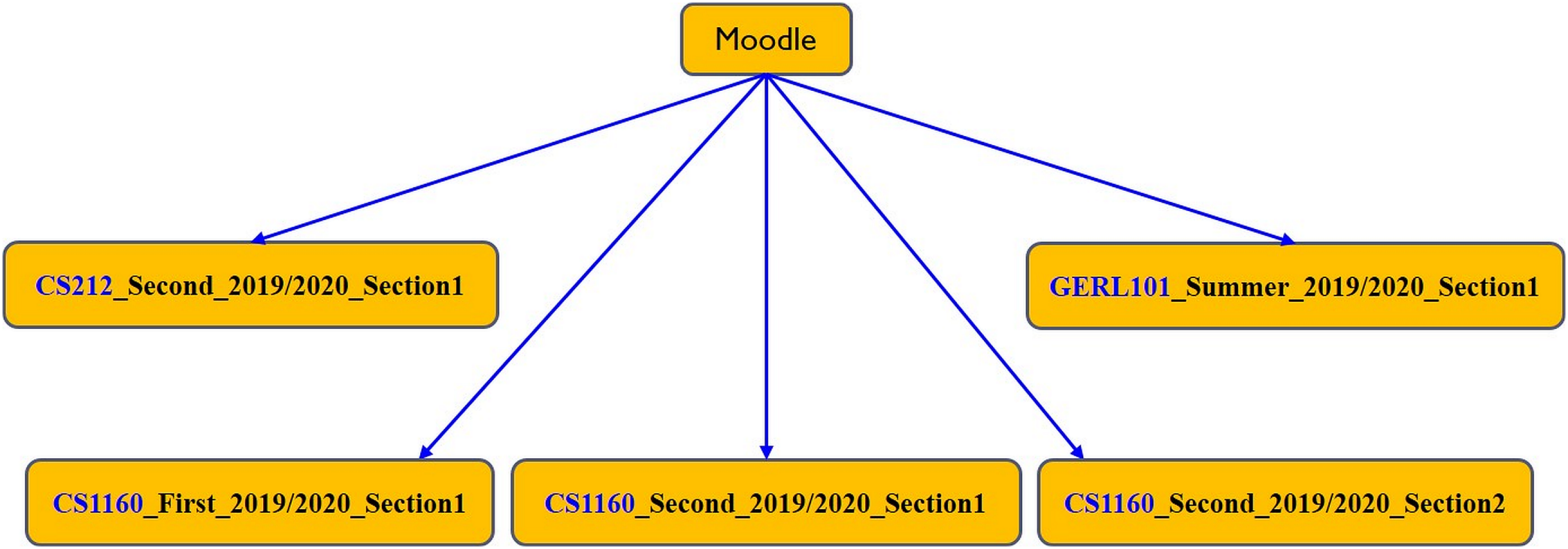
The flat course-section based structure in Moodle.

**Figure 15:**
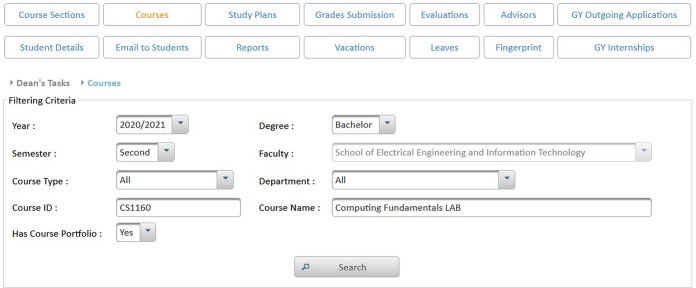
Flexible filtering criteria in MyGJU based on hierarchical information.

The structure of the e-learning data relies on academic information, instructor data, and student data. Therefore, offering the e-learning features in MyGJU, rather than Moodle, allows such modules to gain direct access to the needed data in the SIS, HR, and AIS databases (shown in Fig. 12). Consequently, such tight integration leads to several advantages that are related to:

1. **System setup**: The direct access to all required data reduces the setup steps for the e-learning features, which improves system usability. In that regard, for example, the e-learning admin does not have to repeat the definition of semesters, calendars, and course sections as in the case of Moodle because such information will be immediately available in MyGJU after its definition. Besides, the ability of features such as student attendance, course section homepage, and email to directly access calendars, semesters, schedules, and usernames enables such modules in MyGJU to automate the generation of the attendance days, learning materials weeks, and email recipients, respectively, which relieves users from such tedious tasks.
2. **Information hierarchy**: The data is organized in the MyGJU databases (and hence the MyGJU portal) in a hierarchical manner, which allows advanced filtering by multiple criteria for search or management purposes. For example, the course sections in MyGJU are categorized based on faculties, courses, and semesters as shown in Fig. 13. On the other hand, Moodle only supports a flat course-section based structure (see Fig. 14) that prevents an advanced search for courses and requires enforcing a naming scheme to allow finding course sections that are repeatedly offered every semester such as course CS1160 that has one course section offered on the First 2019/2020 semester, and two course sections offered on the Second 2019/2020 semester. Based on that, MyGJU, unlike Moodle, allows a user to filter courses by faculty, course name, semester, degree, department, course type, and course code as shown in Fig. 15.
3. **Information integrity**: The fact that all data is stored in the database of one system eliminates the possibility of having mismatching data in the different platforms. In that context, mismatching data could be the number of courses, number of course sections, student enrollments, course descriptions, user names, grades, and attendance information.
4. **Information security**: The need to synchronize some of the data in the different platforms based on textual files is eliminated, which reduces the risk of exposing any sensitive data such as grades and account credentials in transit.

### E-learning information reuse

The e-learning related information is associated with either a course or a course section (see Table 1). In this regard, the course information is easier to manage because it can be entered once and then reused when needed in the features of all related course sections. For example, the course description, objectives, learning outcomes, and references can be defined once and then automatically redisplayed in the homepage or syllabus of all respective course sections. Conversely, managing the course section information is relatively harder as it must be entered for each section individually. Especially when tens of course sections for the language courses, for example, are offered every semester and are taught by different instructors. Therefore, reentering similar information, such as exam assessments and weekly learning materials, for each course section can be cumbersome and error-prone. Hence, the common information must be reused, if applicable, to avoid repeating the same work for every course section. Accordingly, MyGJU, unlike Moodle, supports the following capabilities to simplify the setup of the course section related information:

**Table 1:** The scopes of the e-learning related information in MyGJU.

	Course description	Course objectives	Course Learning Outcomes	Course references	Exam assessments	Course folder	Weekly learning materials	Grades
Course level	Yes	Yes	Yes	Yes	No	Yes	No	No
Course section level	No	No	No	No	Yes	No	Yes	Yes

**Figure 16:**
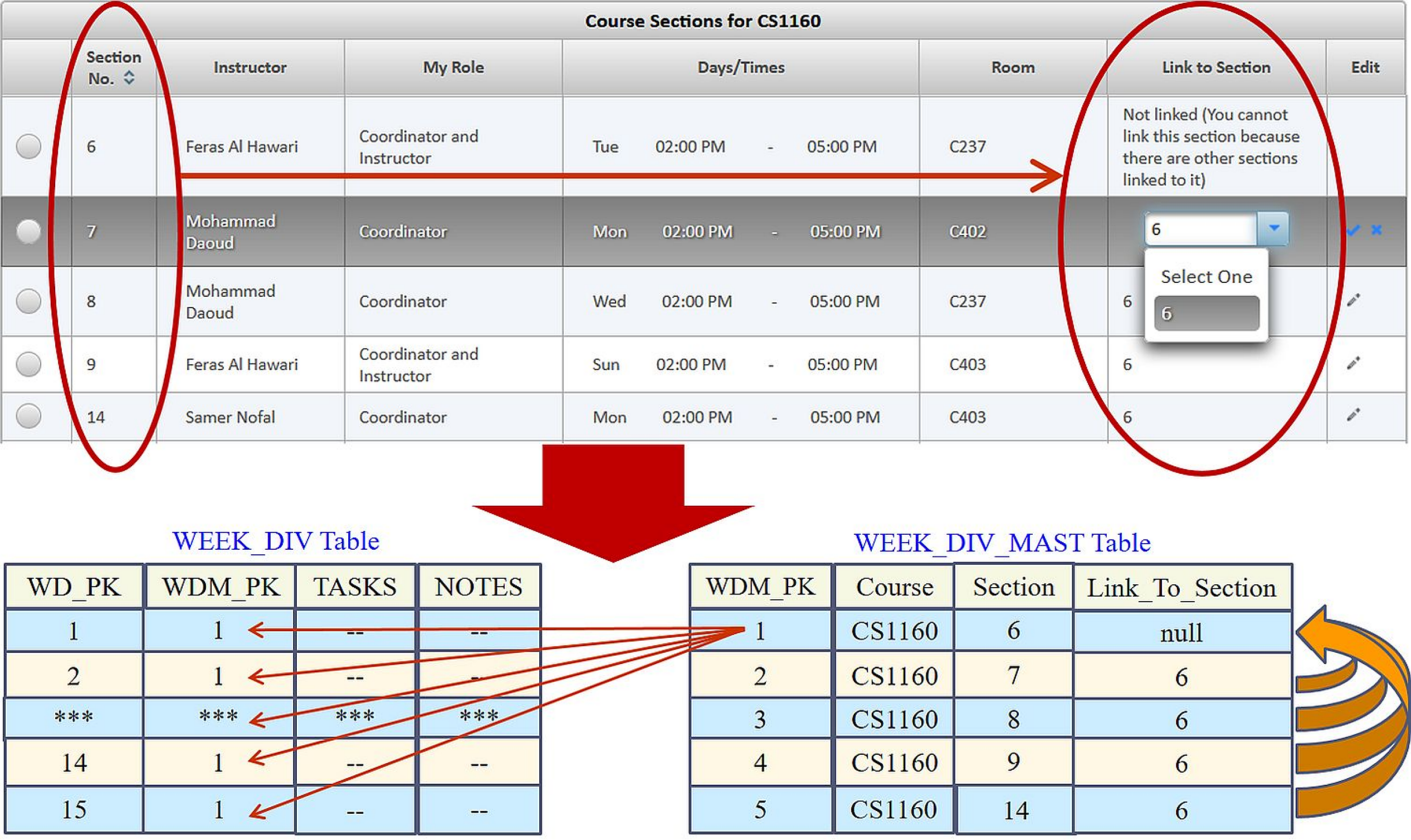
Linking the weekly learning materials of CS1160 course section 6 to course sections 7, 8, 9, and 14.

**Figure 17:**
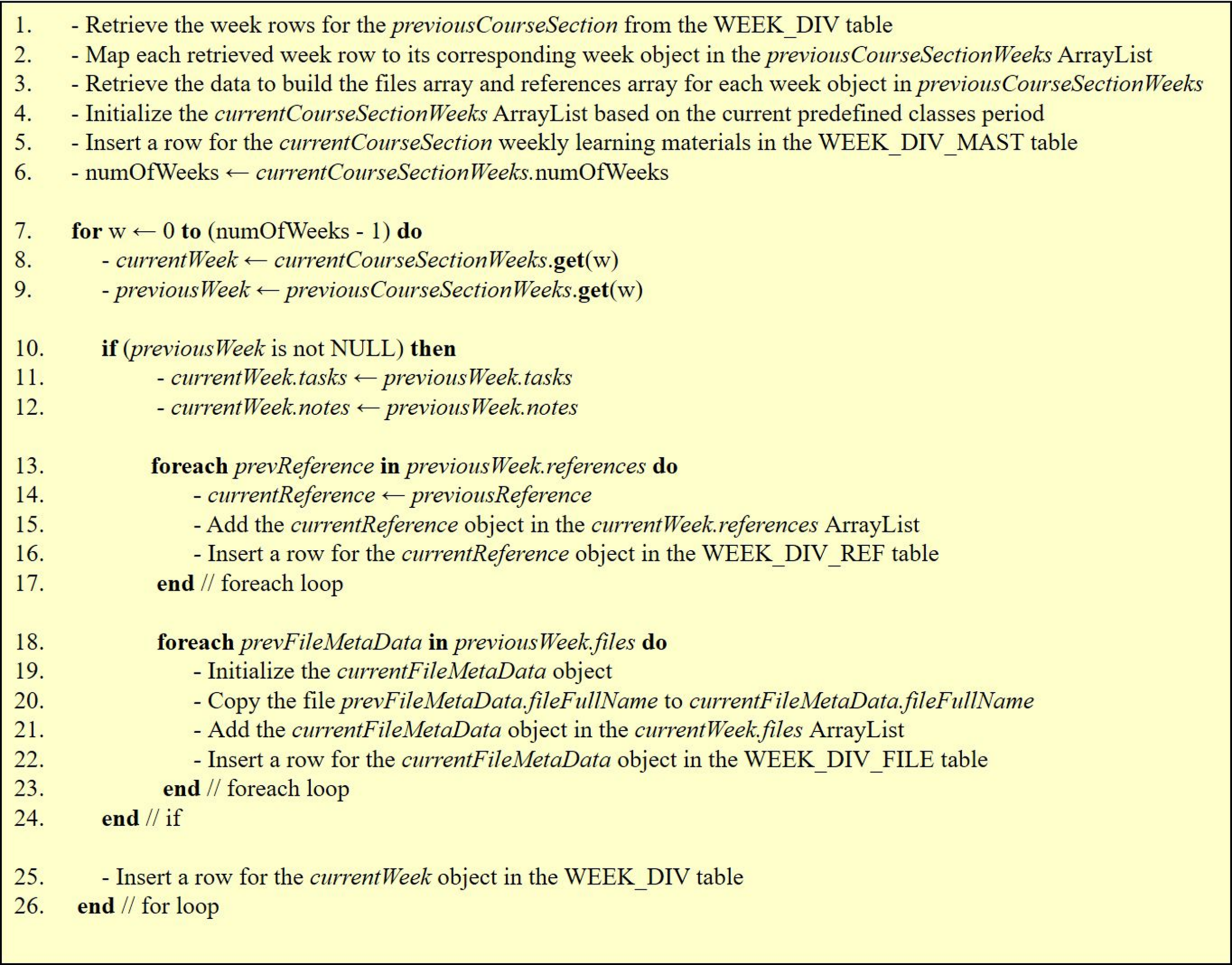
The pseudo code for the copy course section weekly learning materials feature.

1. **Sharing weekly learning materials**: A course coordinator can insert weekly learning materials for a certain course section and then link it to the rest of the related course sections that are offered in the current semester. Noting that when a course section is linked to a shared course section, only the coordinator and instructor of the shared course section may change the weekly learning materials. Hence, a course section should remain unlinked when the instructor desires to personally manage the weekly learning materials. Yet, the fact that all related course sections may share the same weekly learning materials leads to several advantages such as conserving disk space and freeing the course instructors from the needed efforts to make sure that the learning materials are identical for all related course sections. According to the example shown in Fig. 16, course sections 7, 8, 9, and 14 for course CS1160 (Computing Fundamentals Lab) were linked to the weekly learning materials of course section 6, as reflected in the WEEK_DIV_MAST database table. Consequently, those course sections share the 15 weeks that contain the learning materials of course section 6 and are stored in the WEEK_DIV database table. Hence, such a setup eliminated the need to build and save the content of an extra 60 weeks (i.e., the weeks for each of the 4 sections linked to section 6).
2. **Copying weekly learning materials**: The portal also allows initializing the weekly learning materials in a new course section homepage by automatically copying them from a previous course section homepage. Reusing previous weekly learning materials significantly reduces the time and effort that an instructor would usually need to insert the weekly learning materials from scratch. The pseudo code for this feature is shown in Fig. 17.
3. **Copying exam assessments**: The system supports reusing the previously defined exam assessments (if available) for a course as a default. In that case, the previous exam assessments are automatically copied and linked to the course section at hand for reuse or modification.
4. **Reusing files**: The portal also supports uploading a file once to a course folder to be reused many times in all course section homepages (see Fig. 7) or downloaded by any user with read access to that file, which saves disk space and user time.

### E-learning information version control

**Figure 18:**
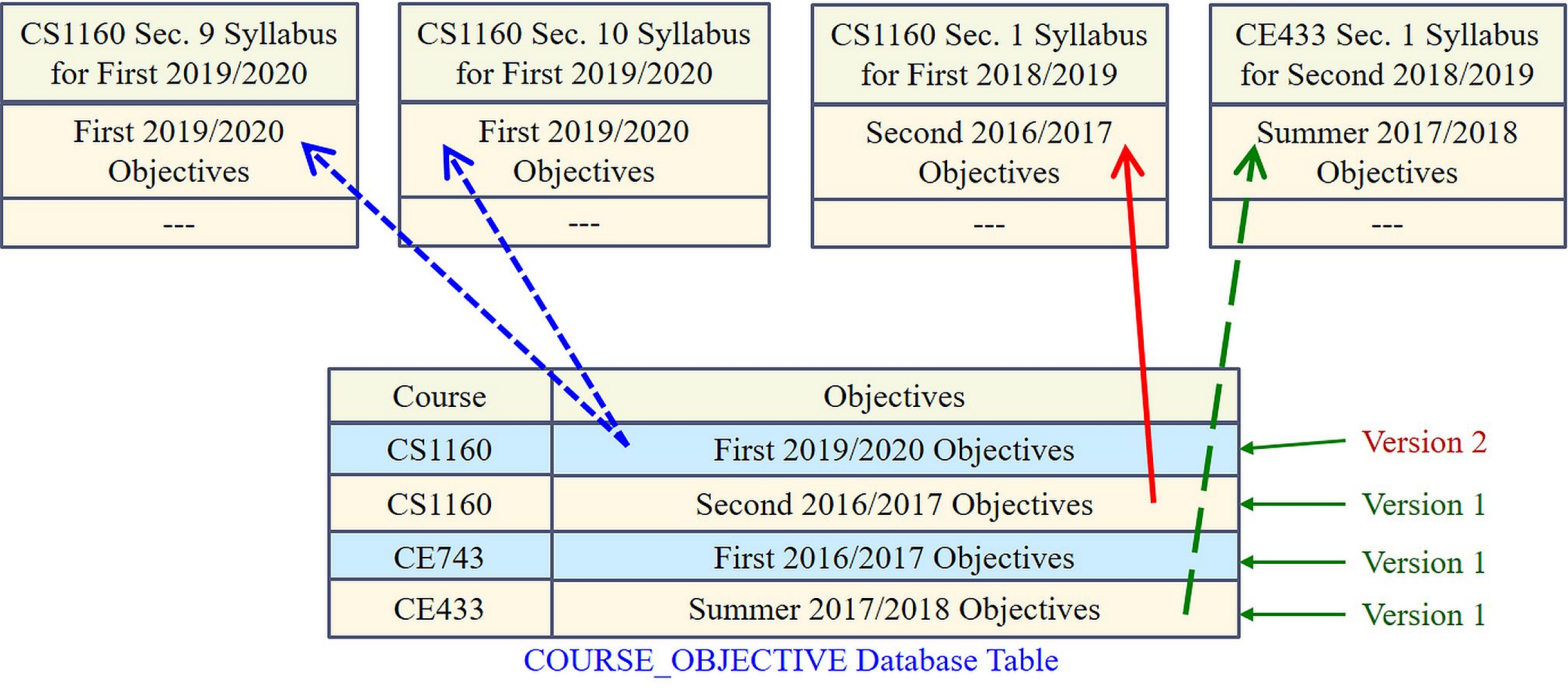
Managing and reusing different versions of course objectives.

The management of changes (version control) to e-learning information is another important aspect that MyGJU, unlike Moodle, automatically and easily supports. In this respect, a semester-based versioning mechanism is adopted in MyGJU to avoid repetitive work and reduce storage demands. Accordingly, once any course-related information is defined in a given semester, there will be no need to redefine it for every later semester as long as the information does not change. In case that information is modified in a new semester, a new version of it that is associated with the modification semester will be generated. Hence, one or more version(s) of any course-related information may exist depending on the number of semesters during which that information was modified. Besides, the most recent version since or before the semester of the information to be constructed is reused when needed (see Fig. 18). On the other hand, only one version of any course section related information is saved in the database. This version is associated with its creation semester, and hence it becomes non-editable when that semester is over.

Based on Fig. 18, course CE433 has one objectives version (i.e., the original Summer 2017/2018 copy), course CE743 also has one objectives version (i.e., the original First 2016/2017 copy), and course CS1160 is associated with two objectives versions (i.e., the original Second 2016/2017 version and the modified First 2019/2020 copy). Accordingly, for example, when the First 2018/2019 semester syllabus for section 1 of course CS1160 is constructed, the related Second 2016/2019 semester course objectives are reused because they are the most recent objectives since or before the desired syllabus semester (i.e., the First 2018/2019 semester). Therefore, the adopted versioning mechanism eliminated the need to redefine and store the objectives of course CS1160, course CE743, and course CE433 in 7 semesters, 9 semesters, and 4 semesters, respectively.

### Flexible role-based user collaboration

MyGJU supports more user roles than Moodle to facilitate a collaborative e-learning process among students, instructors, deans, chairs, and coordinators. It accomplishes that by supporting the following user roles:

1. **Student Role**: This role permits a student to access all basic e-learning features in the MyGJU student view as illustrated in Fig. 2.
2. **Instructor Role**: This role allows instructors to view their schedules, evaluations, and students. In addition, it enables them to manage their course section learning materials, student attendance, and student grades.
3. **Course Coordinator Role**: This role grants a course coordinator the needed privileges to make sure that all weekly learning materials are the same for all shared course sections. An example on such privileges is linking a course section to the weekly learning materials of another section as shown in Fig. 16.
4. **Dean Role**: This role enables school deans to perform all e-learning related tasks pertaining to their schools (see Fig. 15) such as managing course sections, viewing course section homepages, monitoring registration status, viewing student profiles, checking student attendance, approving grades, reviewing instructor evaluations, assigning student advisors, and managing course coordinators.
5. **Chair Role**: This role lets department chairs perform the same tasks that a dean could accomplish but within the scope of their departments.
6. **QA User Role**: This role authorizes Quality Assurance (QA) staff to monitor academic information such as university programs, study plans, course section homepages, course evaluations, student enrollments, student grades, academic staff information, and instructor evaluations. It also allows generating managerial reports to ensure that all GJU programs meet the accreditation requirements.

## Discussion

In this section, the impact of the factors presented in “Methods to Overcome the Moodle Drawbacks” to make certain e-learning features in MyGJU more effective than their counterparts in Moodle is discussed. Moreover, the limitations of the MyGJU e-learning features compared to Moodle are identified. Also, features comparison between MyGJU and other LMSs is conducted.

### Effectiveness of MyGJU modules over their moodle counterparts

Specifically, features comparisons between the course folder, course section homepage, and attendance modules in MyGJU and their counterparts in Moodle are provided in this subsection.

First, a comparison between the course folder feature in MyGJU and the private files folder (Moodle, 2020e) in Moodle is shown in Table 2. Based on that, the folder in MyGJU belongs to a course rather than to an instructor like Moodle, and thus it is accessible to any instructor who teaches the course as well as the dean, chair, and QA officer of the department offering the course. Accordingly, the course folder in MyGJU acts as a course repository and enables educators to share resources, noting that only the owner of a file or subfolder is authorized to share it with others for privacy and copyright related reasons. Besides, the course folder can be accessed from any related course section homepage to select files from it to make them available to students. Additionally, unlike Moodle, MyGJU validates a file content and scans it for viruses to prevent uploading corrupted files to the course folders.

**Table 2:** A comparison between the course folder in MyGJU and the private files folder in Moodle.

	Associated with a Course	Supports permissions and groups	Accessible to all course instructors	Accessible to deans, chairs, and QA users	Accessible from any course section homepage	Supports file upload checks
MyGJU	Yes	Yes	Yes	Yes	Yes	Yes
Moodle	No	For Site Admin Only	Needs Site Admin	No	No	No

Second, the comparison between the course section homepage in MyGJU and its counterpart in Moodle is given in Table 3. Unlike the Moodle Metacourse plugin (Moodle, 2020c) that only allows linking courses for the same instructor and merges the student enrolments in that scenario, MyGJU authorizes a coordinator to link the homepage of a certain course section to other course sections regardless of their instructors and without affecting the student enrolments. Additionally, MyGJU either enables copying previous weekly learning materials homepage to be used as an initial template, or automatically generates empty week divisions according to the semester calendar to be associated with new learning materials. Furthermore, MyGJU permits reusing course data such as description, objectives, learning outcomes, and references in all related course section homepages. Moreover, it allows linking a week division in a course section homepage to course references and course folder files. Besides, course section homepages are accessible by related managers for evaluation and reporting purposes.

**Table 3:** A comparison between the course section homepage in MyGJU and its counterpart in Moodle.

	Awareness of calendar	Reuse previous weekly learning materials	Linkable to any course section	Inherits course data	Links week to references	Reuses files from course folder	Accessible to deans, chairs, and QA users
MyGJU	Yes	Yes	Yes	Yes	Yes	Yes	Yes
Moodle	No	No	No	No	No	No	No

Third, a comparison between the student attendance flow in MyGJU and the attendance activity (Moodle, 2020a) in Moodle is illustrated in Table 4. Unlike Moodle, the attendance flow in MyGJU neither requires installation nor setup. On the contrary, it is much easier to use because it automatically generates the attendance days based on its knowledge of the course section days and the semester period. Moreover, MyGJU supports taking the attendance for multiple days from one screen in contrast with Moodle that requires switching between the management screen and attendance screen several times to record the attendance for each day separately. Also, MyGJU can identify attendance violations based on its knowledge of the defined thresholds in the GJU regulations. Besides, the student attendance information is available to managers for evaluation and further action.

**Table 4:** A comparison between the attendance flow in MyGJU and its counterpart in Moodle.

	Needs installation	Requires setup	Awareness of calendar and schedule	Supports taking the attendance of multiple days	Awareness of GJU regulations	Ability to report violations	Accessible to deans, chairs, and registrars
MyGJU	No	No	Yes	Yes	Yes	Yes	Yes
Moodle	Yes	Yes	No	No	No	No	No

Nevertheless, the fact that Moodle is not integrated with the SIS database necessitates extra setup steps each semester such as uploading the offered course sections from MyGJU to Moodle, importing student enrolments from MyGJU to Moodle, and exporting quiz grades from Moodle to MyGJU. Besides, unlike MyGJU, Moodle does not recognize the university organizational structure, the semester in which the course sections are offered, or the schools that offer the course sections. Therefore, Moodle has to organize the course sections in a flat structure and without any associations, which hinders information reuse, sharing, and version control. The previous fact does not allow Moodle to support advanced user roles to permit deans, chairs, and coordinators to access the resources of their schools for either evaluation, approval, analysis, or reporting.

### Limitations of MyGJU compared to Moodle

The fact that Moodle is open source enables developers to access, modify, and enhance its source code. Hence, the scale and pace of introducing new features in Moodle can be much bigger and faster than that in MyGJU. Moreover, unlike Moodle, MyGJU does not have any support for external plugins that enable anyone to share a plugin with the community. In addition, the MyGJU features are customized to satisfy the GJU needs rather than fulfilling the general needs of any institution as in the case of Moodle. Also, MyGJU does not support some of the interactive activity tools that are available in Moodle such as quizzes, lessons, assignments, chats, and forums.

In the short term, and as a workaround for the current MyGJU limitations, the web-based examination management system (Al-Hawari et al., 2019) that is developed in-house as well as Moodle are utilized at the GJU to conduct online assignments, quizzes, and examinations. Furthermore, the GJU instructors could take advantage of the available Microsoft 365 (Microsoft, 2021) subscription to live stream, and record, lectures via MS Teams. Yet, MS Teams might be used for group chats and document sharing. Not to mention, the hyperlinks to the recorded lectures are added in the corresponding week division in the related course section homepage to enable the students to access all course learning materials from MyGJU.

### Features comparison between MyGJU and existing in-house LMSs

As mentioned in “Literature Review”, several in-house LMSs were introduced in the studies in Abdulazeez & Zeebaree (2018), Alomran et al. (2020), Alseelawi et al. (2020), Aybay & Dag (2003), Elkhateeb, Shehab & El-Bakry (2019), Jung & Huh (2019), Wu et al. (2018) and Yueh & Hsu (2008). Suitably, an e-learning related comparison between MyGJU and the aforementioned systems is shown in Table 5. Accordingly, it is obvious that the main contributions of this work are related to the following e-learning capabilities: coordination, attendance, course folder, course section homepage, learning materials, student tracking, information reuse, version control, and advanced user roles. Particularly, none of the existing systems in Table 5 have discussed topics like coordination, course folder, and version control. Besides, at most one of the related systems supported syllabus (Yueh & Hsu, 2008), student tracking (Aybay & Dag, 2003), information reuse (Yueh & Hsu, 2008), and advanced user roles (Abdulazeez & Zeebaree, 2018). Further, at most two of the existing systems mentioned attendance (Alomran et al., 2020; Aybay & Dag, 2003) and weekly learning materials homepage (Alseelawi et al., 2020; Yueh & Hsu, 2008). Similarly to this work, several researchers asserted the importance of having features such as integration with SIS, grades, rich content, files, email, and survey in LMSs. As it happens, some of the weaknesses of the aforesaid systems intersect with the noticed Moodle drawbacks, in fact that asserts the importance of this research to propose solutions for such issues pertaining to some LMSs. On the other hand, unlike various systems, MyGJU is still missing some of the needed activity tools such as quizzes and discussion forums.

**Table 5:** Features comparison between MyGJU and existing in-house LMSs.

	MyGJU	(Alomran et al., 2020)	(Alseelawi et al., 2020)	(Jung & Huh, 2019)	(Elkhateeb, Shehab & El-Bakry, 2019)	(Abdulazeez & Zeebaree, 2018)	(Wu et al., 2018)	(Yueh & Hsu, 2008)	(Aybay & Dag, 2003)
Integration with SIS	Yes	Yes	No	No	No	Yes	No	Yes	Yes
Coordination	Yes	No	No	No	No	No	No	No	No
Attendance	Yes	Yes	No	No	No	No	No	No	Yes
Assessment & Grades	Yes	Yes	No	No	Yes	Yes	No	Yes	Yes
Course Folder	Yes	No	No	No	No	No	No	No	No
Syllabus	Yes	No	No	No	No	No	No	Yes	No
Weekly Learning Materials Homepage	Yes	No	Yes	No	No	No	No	Yes	No
Rich Content	Yes	No	No	Yes	No	No	Yes	Yes	No
Files	Yes	Yes	Yes	Yes	Yes	Yes	Yes	Yes	Yes
Quizzes	No	Yes	No	No	Yes	No	Yes	No	Yes
Forum	No	No	No	No	Yes	No	Yes	Yes	Yes
Student Tracking	Yes	No	No	No	No	No	No	No	Yes
Email	Yes	Yes	No	No	Yes	Yes	No	Yes	Yes
Survey	Yes	Yes	No	No	Yes	No	No	Yes	No
Information Reuse	Yes	No	No	No	No	No	No	Yes	No
Version Control	Yes	No	No	No	No	No	No	No	No
Advanced User Roles	Yes	No	No	No	No	Yes	No	No	No

## Validation and results

The MyGJU deployment results and user survey outcomes are presented next.

### MyGJU deployment results

**Figure 19:**
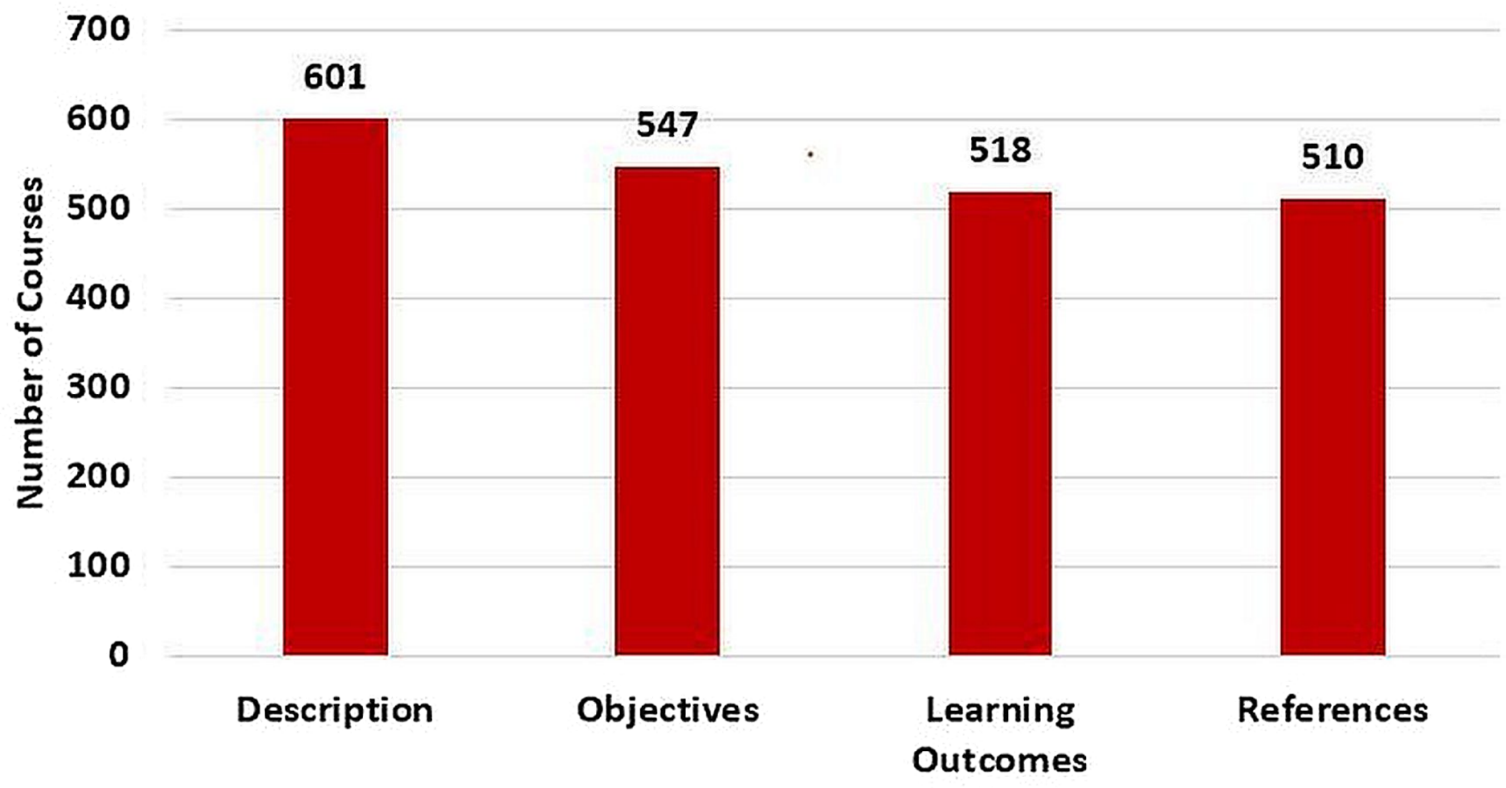
Number of courses with description, objectives, learning outcomes, and references.

Based on Fig. 19, the numbers of courses with defined description, objectives, learning outcomes, and references are 601, 547, 518, and 510, respectively. Noting that there is a total of 1,616 graduate and undergraduate courses at GJU.

**Figure 20:**
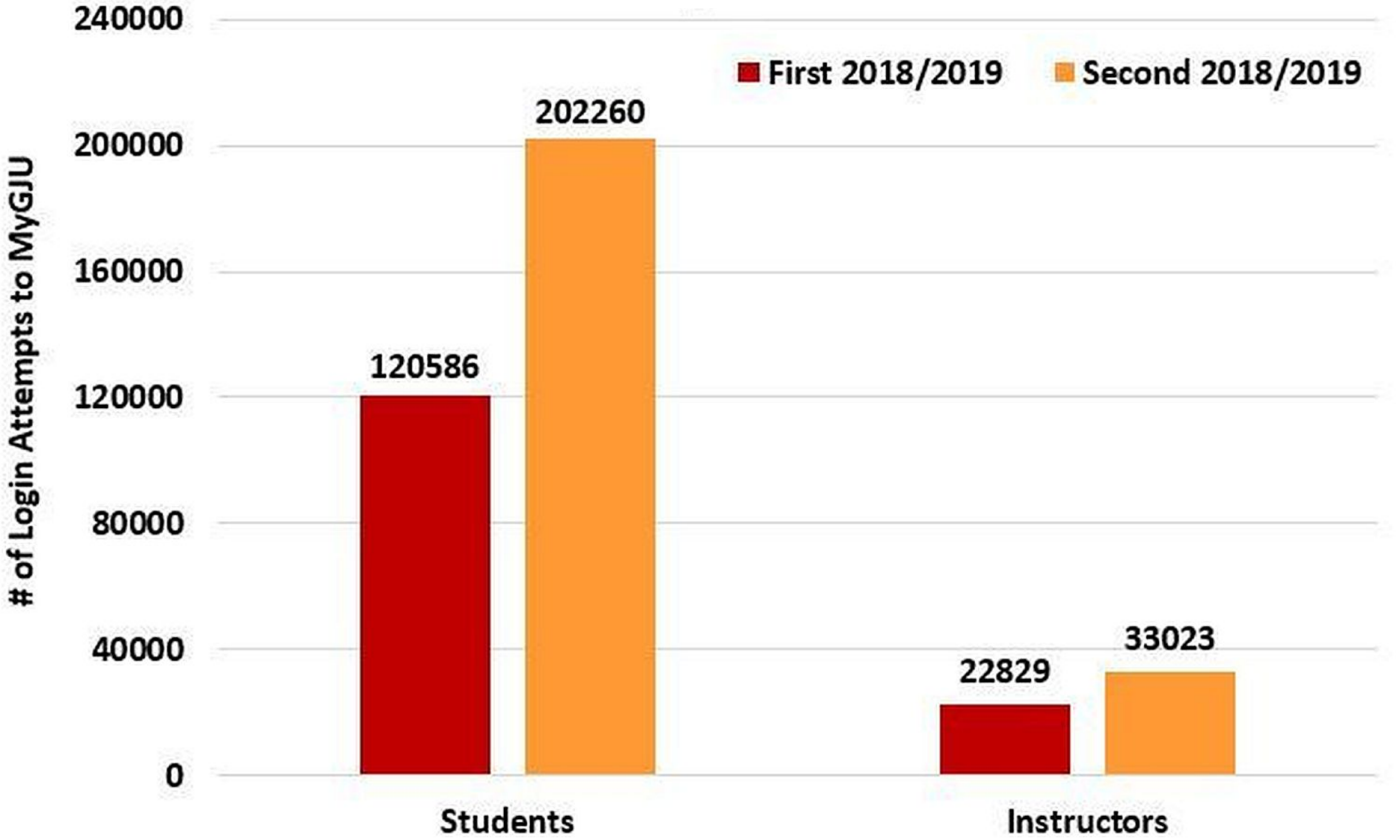
Number of login attempts to MyGJU in the First 2018/2019 and Second 2018/2019 semesters.

The course folder, course section homepage, and file upload features were introduced in MyGJU in the Second 2018/2019 semester. In light of that, it was expected to see instructors and students login more frequently to MyGJU during that semester, as opposed to the previous semester, to manage course section homepages and access course learning materials, respectively. As expected, the user login attempts to MyGJU shown in Fig. 20 indicate a huge increase in traffic to MyGJU during the Second 2018/2019 semester. Accordingly, the total number of student and instructor login attempts to MyGJU increased by 67.7% and 44.6%, respectively, from the First 2018/2019 semester (i.e., the semester before launching those features) to the Second 2018/2019 semester.

**Figure 21:**
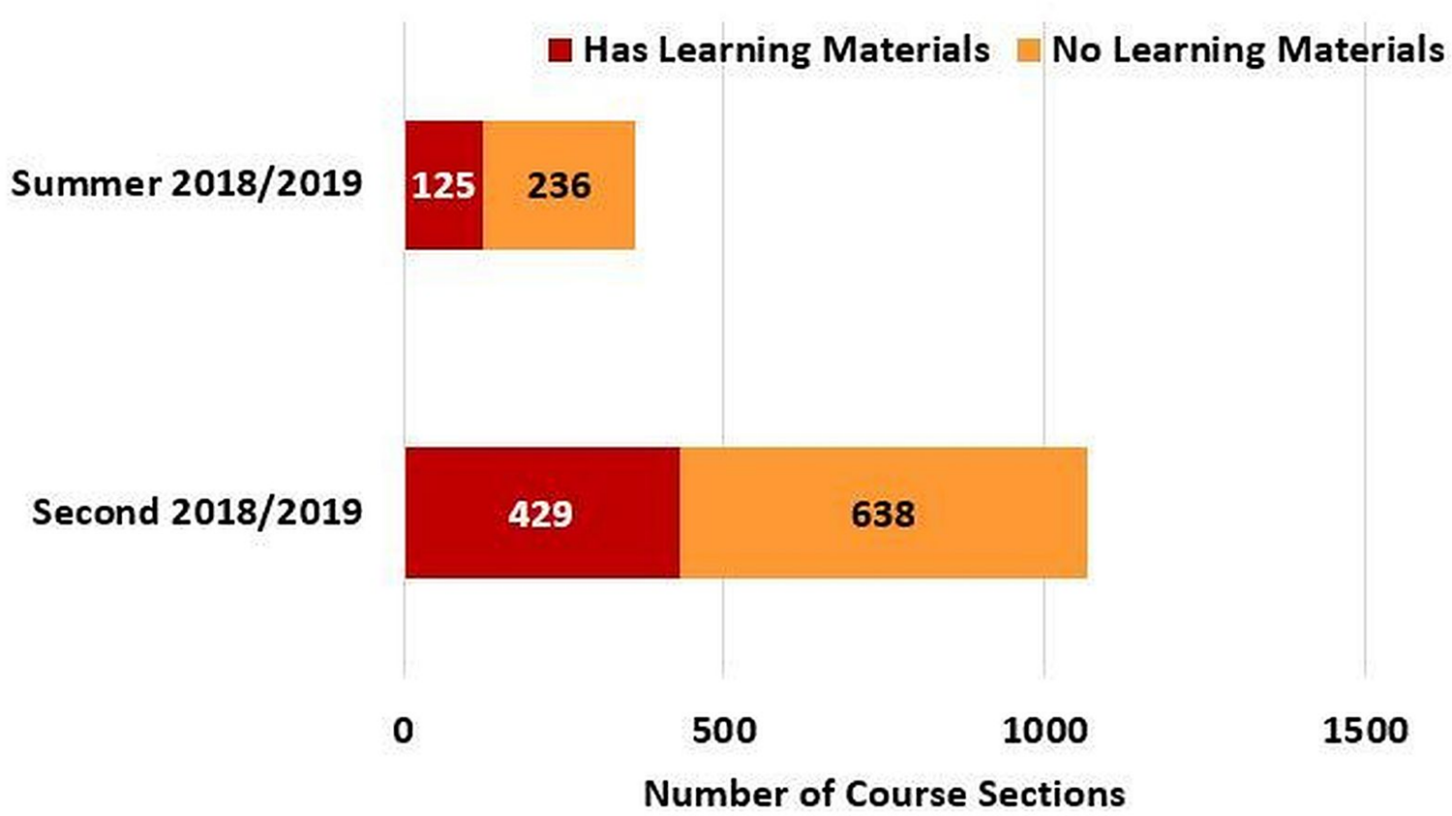
Course sections with and without weekly learning materials in the Second 2018/2019 and Summer 2018/2019 semesters.

**Figure 22:**
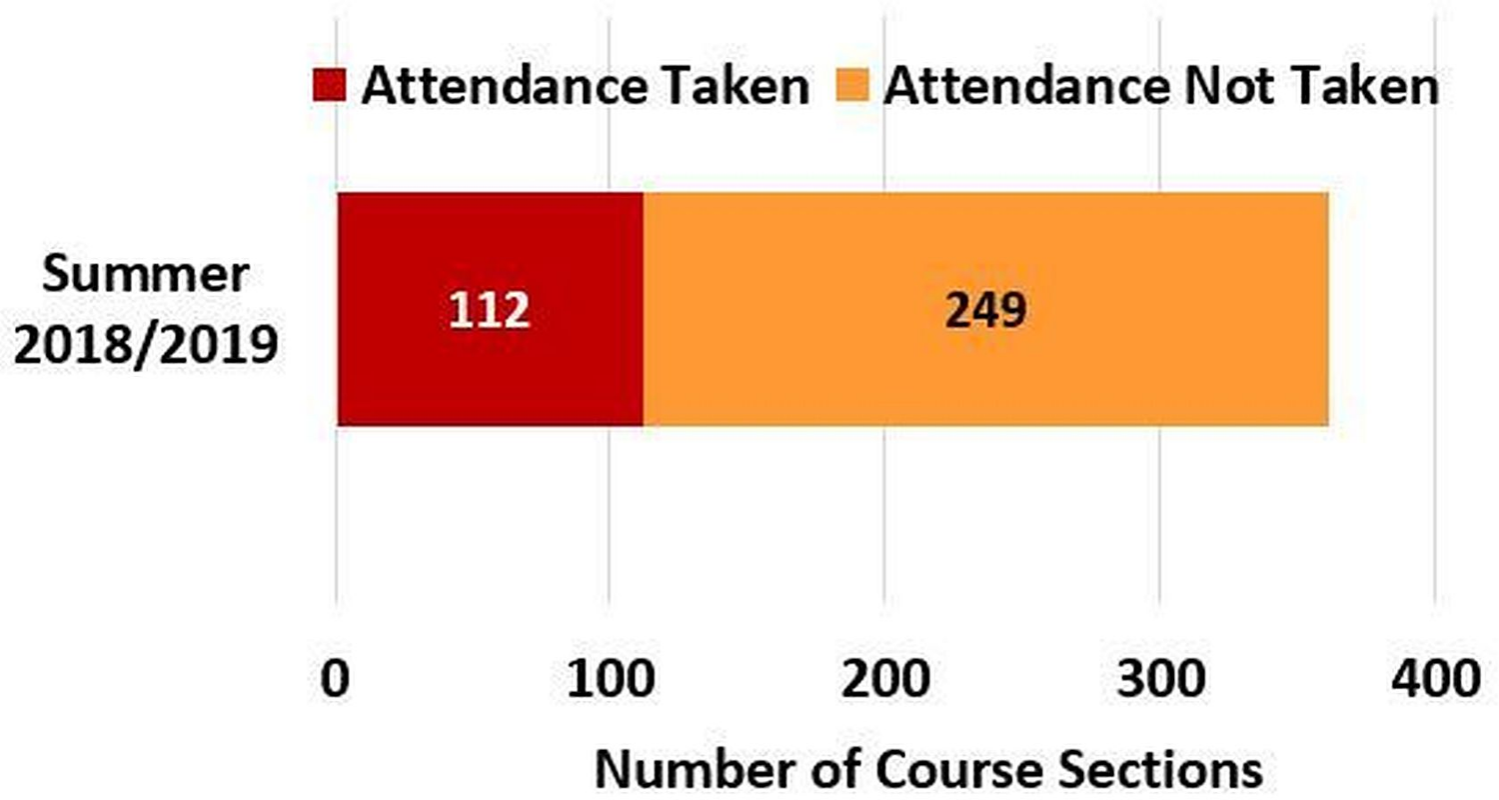
Course sections with attendance taken or not taken in the Summer 2018/2019 semester.

**Figure 23:**
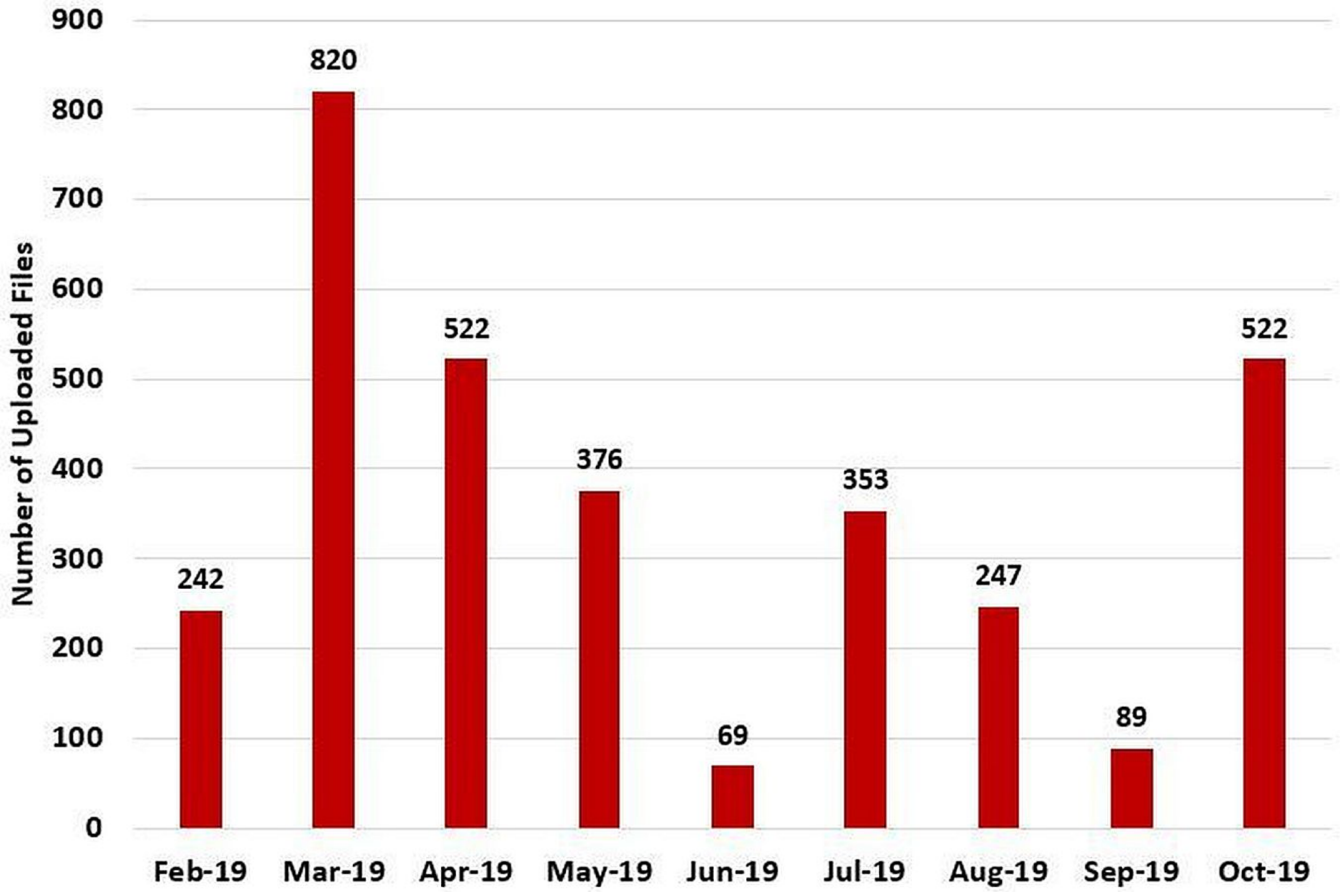
The total uploaded learning documents in MyGJU from February 2019 to October 2019.

Besides, based on Fig. 21, the number of offered course sections in the Second 2018/2019 semester and the following Summer 2018/2019 semester were 1067 and 361, respectively. Further, 40.2% and 34.6% of the total course sections in the Second 2018/2019 semester and the Summer 2018/2019 semester, respectively, contained learning materials. Not to mention, according to Fig. 22, the students’ attendance was taken for 31% of the 361 course sections that were offered in the Summer 2018/2019 semester (i.e., the semester in which the student attendance management flow was launched in MyGJU). Furthermore, according to Fig. 23, as many as 3240 files were uploaded in MyGJU since that feature was introduced in the Second 2018/2019 semester, which started on February 2019, till October 2019 (i.e., the start of the First 2019/2020 semester).

In that regard, the recorded activities illustrate that the new e-learning features are engaging and useful because many users are utilizing them in the teaching and learning processes. However, such numbers are expected to improve further when more users become familiar with, and start using, such capabilities. Not to mention, the role of the QA officers to market, monitor, and enforce the completion of all course section homepages to meet the accreditation requirements, hence that can be another important factor to increase the reported numbers.

### User survey results

**Figure 24:**
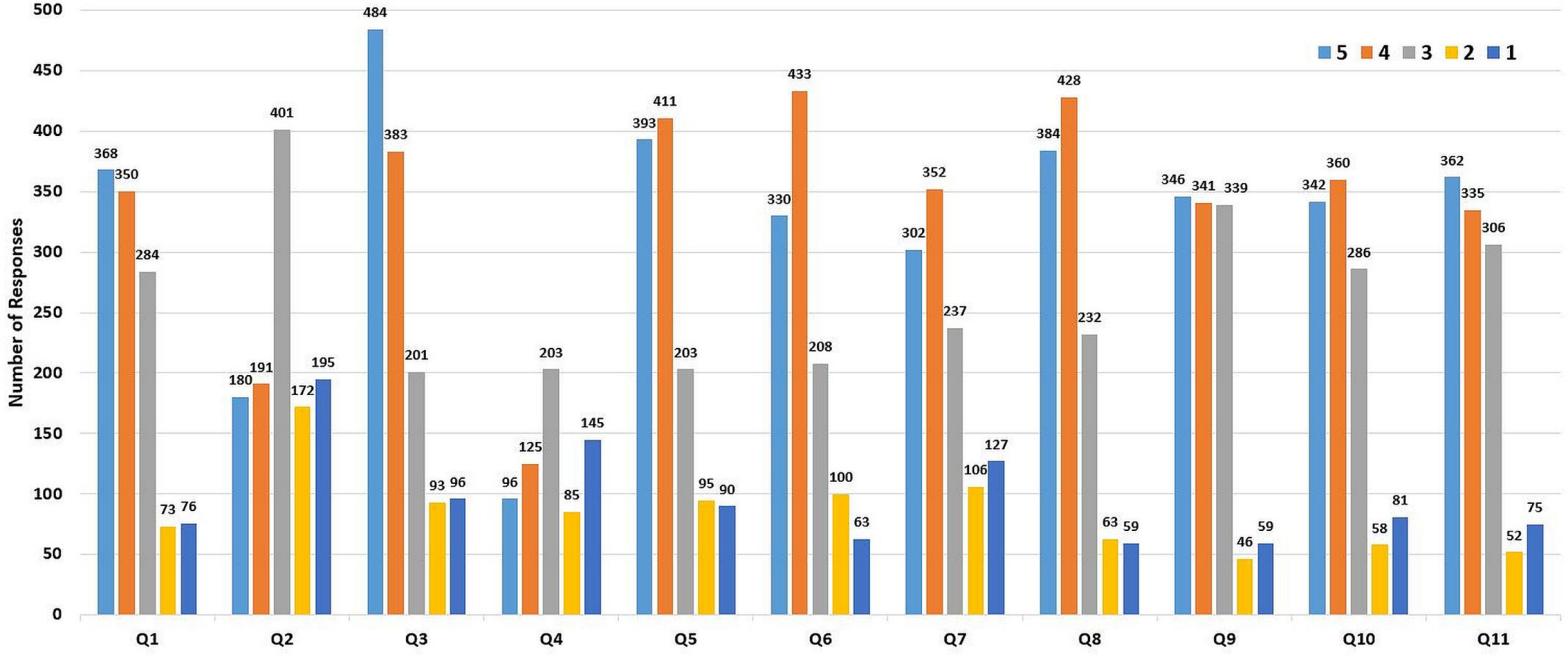
The different total responses to each question in Table 6.

A total of 1,325 users (169 instructors and 1,156 students) participated in a survey that was conducted for 2 weeks at the beginning of the First 2019/2020 semester to assess user satisfaction regarding making MyGJU as a FPOC for basic e-learning tasks. The survey questions are found in Table 6, whereas the results are shown textually in Table 6 and graphically in Fig. 24. According to Table 6, each question has five Likert-scale based answers as follows: Strongly Agree, Agree, Neutral, Disagree, and Strongly Disagree. While the aforesaid five answers map to 5 points, 4 points, 3 points, 2 points, and 1 point, respectively.

**Table 6:** The user survey results.

#	Question	Answers					Total answers	Average score	Standard deviation
		5	4	3	2	1			
Q1	I prefer to keep using MyGJU for most e-learning related tasks	368	350	284	73	76	1,151	3.748045	0.669671
Q2	I prefer to go back to Moodle for most e-learning related tasks	180	191	401	172	195	1,139	2.990342	0.361220
Q3	I use the e-learning features in MyGJU at least once a week	484	383	201	93	96	1,257	3.848051	0.788707
Q4	I still use Moodle at least once a week	96	125	203	85	145	654	2.911315	0.320885
Q5	I use the course portfolio in MyGJU for course slides and materials	393	411	203	95	90	1,192	3.773490	0.718329
Q6	I obtain the syllabus from the course portfolio in MyGJU	330	433	208	100	63	1,134	3.764550	0.698655
Q7	I use the attendance feature in MyGJU	302	352	237	106	127	1,124	3.530249	0.576605
Q8	The course portfolio feature in MyGJU is easy to use	384	428	232	63	59	1,166	3.870497	0.748611
Q9	The attendance screens in MyGJU are easier to use than those in Moodle	346	341	339	46	59	1,131	3.768347	0.665587
Q10	The attendance report in MyGJU is informative	342	360	286	58	81	1,127	3.731145	0.660656
Q11	Chatting with peers in MyGJU will be useful	362	335	306	52	75	1,130	3.758407	0.674465

Based on the responses to question 1, about 62% of the users strongly agreed or agreed to keep using MyGJU for the basic e-learning related tasks. Whereas, according to the responses to question 2, only 33% of the users strongly agreed or agreed to utilize Moodle for most e-learning related tasks. Also, according to question 3, around 69% of the responders strongly agreed or agreed that they use the e-learning features in MyGJU at least once a week. On the other hand, based on question 4, just 34% of the users strongly agreed or agreed that they are using the e-learning features in Moodle at least once a week. According to questions 5–7, the percentage of users who strongly agreed or agreed that they use MyGJU to download course materials, obtain course syllabus, and check attendance were 67%, 67%, and 58%, respectively. Based on questions 8–10, the percentage of users who strongly agreed or agreed that the course portfolio feature in MyGJU is easy to use, the attendance screens in MyGJU are easier to use than those in Moodle, and the attendance report is informative were 70%, 61%, and 62%, respectively. Further, according to the responses to question 11, about 62% of the users strongly agreed or agreed that having a chat feature in MyGJU will be useful. Yet, the standard deviation for each question is not large, which asserts the accuracy of the reported results.

Accordingly, the user survey results confirm that the majority of users preferred to use MyGJU, as opposed to Moodle, for basic e-learning tasks. Further, since the users were used to Moodle the past 10 years, it is expected that the acceptance of MyGJU will improve much more as the users get more accustomed to the new e-learning features in MyGJU.

## Conclusion and future work

The use of e-learning tools in higher education institutions has become imperative to empower teaching and learning. However, delivering effective e-learning features to users can be challenging, especially when an institution relies on more than one platform to provide such services. In that regard, this research proved that the in-house development of the basic e-learning features within MyGJU to make it a FPOC, as opposed to Moodle, to accomplish daily educational tasks has gained quick acceptance among users due to the following reasons:

- Performing the majority of the daily e-learning tasks from a customized web-based university portal is more convenient than doing so from multiple platforms. Examples on such tasks are: managing learning materials, taking student attendance, entering exam grades, and sending emails to peers.
- Storing the data in the databases of one platform eliminated the need to synchronize information every semester between different platforms. Hence, that simplified system setup, guaranteed data integrity, and maintained security.
- Organizing the data in a hierarchical manner in MyGJU allowed performing advanced filtering by multiple criteria for search and management purposes.
- The capabilities to compose some information once and then reusing them many times eliminated a lot of the repetitive work and reduced storage demands. For example, the course data such as name, description, objectives, learning outcomes, and references can be defined once and then reused in any related course sections. Further, a file can be uploaded once to a course folder and then linked to many week divisions in the course section homepages. Besides, the weekly learning materials for a course section may be linked to several other course sections.
- The features to copy the learning materials or exam assessments of a previous course section to the homepage of a current course section reduced the time and effort that an instructor would usually need to insert that information from scratch.
- Supporting additional roles and permissions enabled deans, chairs, course coordinators, and QA officers to access all the required information to perform either coordination, evaluation, approval, analysis, or reporting tasks.
- The ability to develop the needed features in-house facilitated the customization of the tools, for example, to make them easier to use, to perform checks according to university regulations, to automate a series of tedious steps, or to generate personalized reports.

Nevertheless, the reliance on MyGJU to offer the basic e-learning features does not fully eliminate the need for having a LMS that complements the available MyGJU capabilities with missing tools or advanced features that are not as frequently used. For example, Moodle can still coexist with MyGJU to conduct online exams as well as to utilize some of its free external plugins like the live video streamer and the plagiarism checker.

In regard to future work, the focus will be on complementing the offered modules in MyGJU with activity tools like chat, forums, and lessons to facilitate interaction among all collaborators in the educational process from one place. Further, the available online exams system can be integrated with MyGJU to utilize it for conducting assignments, quizzes, and exams.

## Supplemental Information

10.7717/peerj-cs.498/supp-1 Supplemental Information 1 Copy of Questionnaire.

10.7717/peerj-cs.498/supp-2 Supplemental Information 2 Raw data for survey in Table 5.

## Additional Information and Declarations

### Data Availability

The following information was supplied regarding data availability: Raw data are available as Supplemental Files.
