# Supplementary figures and images for: Methods to achieve effective web-based learning management modules: MyGJU versus Moodle
Source: PeerJ Comput Sci. 2021 Apr 16;7:e498. doi: 10.7717/peerj-cs.498 (PMC8056247; doi:10.7717/peerj-cs.498)

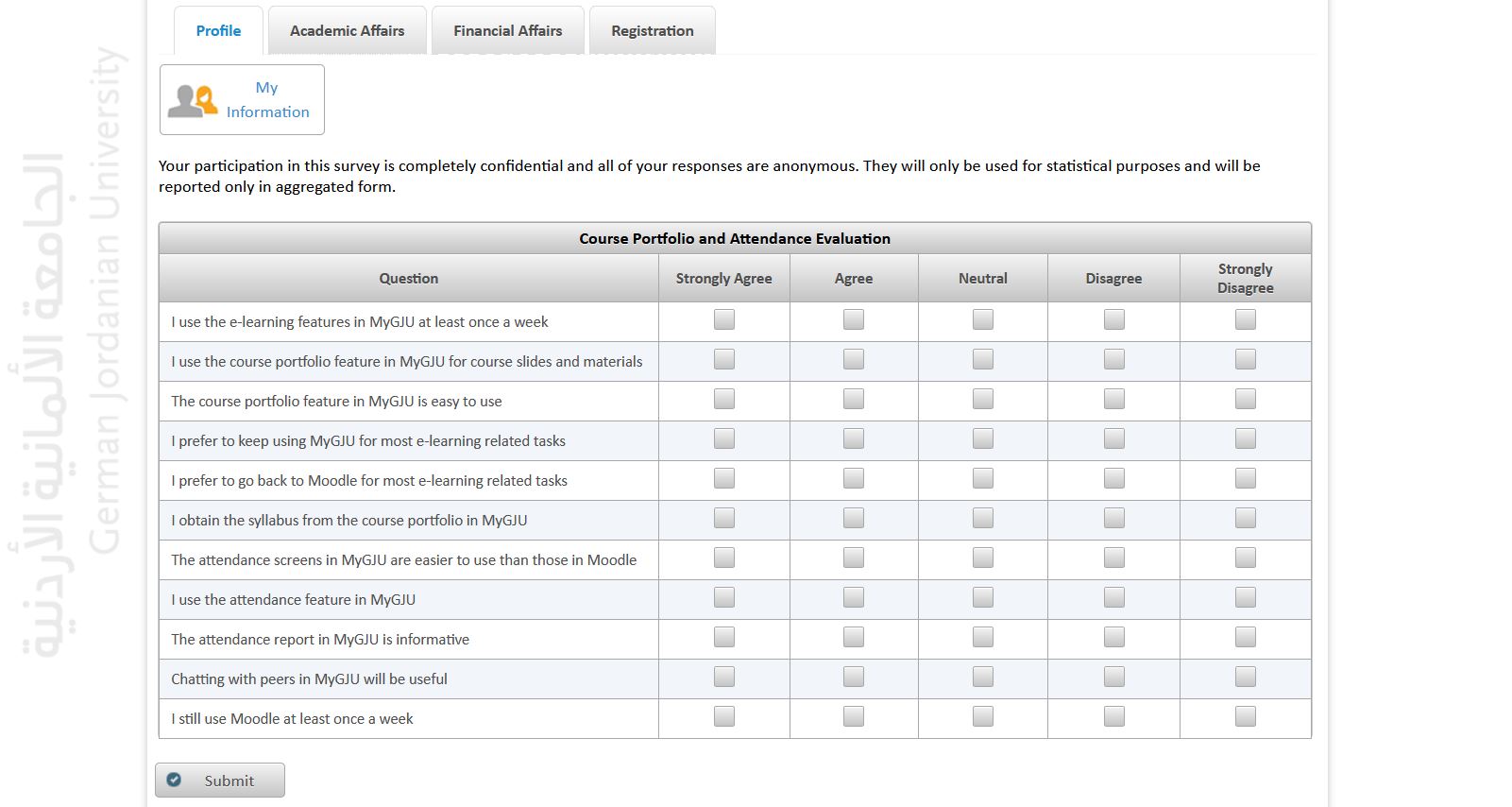

Supplement: Supplemental Information 1 [file peerj-cs-07-498-s001.jpg]
